# The Associations of Air Pollution Mixture Exposure with Plasma Proteins in an Elderly U.S. Panel

**DOI:** 10.1021/acs.est.5c03052

**Published:** 2025-07-24

**Authors:** Ziyin Tang, Ying Wang, Jeremy A. Sarnat, W.Ryan Diver, Todd M. Everson, Emily Deubler, Youran Tan, Stephanie M. Eick, Aparna H. Kesarwala, Michelle C. Turner, Carmen J. Marsit, Mattias Johansson, Hilary A. Robbins, Donghai Liang

**Affiliations:** † Gangarosa Department of Environmental Health, Rollins School of Public Health, 25798 Emory University, Atlanta, Georgia 30322 United States; ‡ Department of Population Science, 1369 American Cancer Society, Atlanta, Georgia 30303, United States; § Department of Radiation Oncology, Emory University School of Medicine, Atlanta, Georgia 30322, United States; ∥ Barcelona Institute for Global Health (ISGlobal), Barcelona 08036, Spain; ⊥ Universitat Pompeu Fabra (UPF), Barcelona 08018, Spain; # Genomic Epidemiology Branch, 56140 International Agency for Research on Cancer, Lyon 69366, France

**Keywords:** air pollution, long-term exposure, mixture, proteins, immune responses, inflammation, signaling

## Abstract

The impact of air pollution exposure on circulating proteins remains underexplored, particularly in vulnerable elderly populations. This study investigated the individual and joint effects of air pollutants on circulating proteins in 208 elderly participants from the Cancer Prevention Study-II Nutrition Cohort. Prediagnostic plasma samples were collected (1998–2001), and 484 proteins were measured using the Olink platform. Annual average exposures to six air pollutants in the calendar year of blood draw were estimated. We used linear regression for individual pollutants and quantile g-computation for mixture effects, adjusting for confounders, considering multiple comparison correction, and testing interactions with smoking status. Pathway enrichment and protein–protein interaction analyses were conducted for the associated proteins. We identified 167 distinct proteins associated with individual pollutants or mixtures (*p* < 0.05), including 15 meeting a false discovery rate <0.2. IL32, ADAM15, and IL8 demonstrated consistent negative associations with ≥4 exposure metrics. Twenty proteins were associated with both mixtures and individual air pollutants with consistent effect directions. These proteins were enriched in pathways linked to immunity and signaling. Stratified analyses revealed differing associations with 99 proteins between current and former smokers. The findings offer valuable insight into the chronic biological response in plasma protein levels to air pollution exposure.

## Graphical abstract

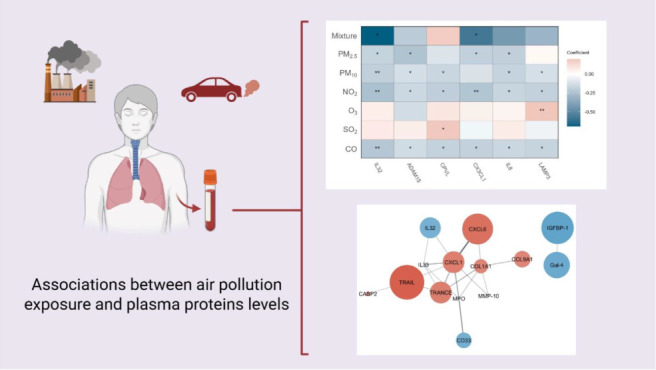

## Introduction

Exposure to ambient air pollutants, including fine and coarse particulate matter (PM_2.5_ and PM_10_), nitrogen dioxide (NO_2_), ozone (O_3_), sulfur dioxide (SO_2_), and carbon monoxide (CO), has been associated with various adverse health outcomes including lung cancer.^1–4^ The primary mechanisms underlying air pollution-associated health effects are suggested to involve oxidative stress, inflammation-related cascades, and genotoxicity.^5–7^ However, the underlying biological mechanisms, including detailed molecular events and biological pathways, remain underexplored. Historically, a few targeted inflammation and oxidative stress biomarkers related to these processes, such as 8-oxoguanine, several interleukins (ILs), and tumor necrosis factor-α (TNF-α), have been utilized in air pollution epidemiological studies.^7,8^ Nevertheless, the reported associations of air pollution exposure with these biomarkers have been inconsistent and not robust.^7,8^ To advance the understanding of the relevant biological mechanisms associated with air pollution-related etiologies and improve exposure assessment, the development of specific and sensitive biomarkers is essential.

High-throughput omics approaches have demonstrated great potential in addressing these gaps by uncovering molecular signals and biological pathways related to complex environmental exposures like air pollution.^9^ Previously, our group conducted several air pollution metabolomics analyses.^10–14^ However, metabolomics only provides a snapshot of the end products of cellular processes.^15^ Proteomics, the comprehensive study of proteins within specific biosamples, reveals the functional output of gene expression and cellular functions.^16^ Proteins are involved in nearly all biological reactions and interactions within living organisms.^17^ A comprehensive analysis of the associations between air pollution exposure and proteins will offer valuable insights into how air pollution exposure may perturb these critical biological functions and which proteins may play critical roles within these processes.

A number of recent epidemiological studies have assessed the associations between air pollution exposure and protein levels in specific biospecimens.^18–29^ These studies primarily focused on particulate matter (PM) and NO_2_ exposure, while other air pollutants remain underexplored. Additionally, in a real-world setting, people are exposed to multiple air pollutants simultaneously. Previous studies typically evaluated the effects of individual air pollutants on proteins separately. This approach overlooks the potential joint effects of air pollution mixtures and the high correlations among these pollutants, which may not accurately reflect real-world exposure scenarios. Moreover, existing air pollution-protein investigations generally analyzed a limited panel of proteins (i.e., a few to dozens), primarily focusing on inflammatory cytokines and adipokines with inconsistent findings. The inconsistencies may be due to differences in study design, study populations, biosample types, exposure assessment methods, exposure time windows, statistical approaches, and residual confounding. Lastly, relatively few investigations have been conducted in elderly populations, who are particularly susceptible and vulnerable to elevated air pollution exposures.^22,23,29^ Age-related physiological declines, such as reduced lung, cardiovascular, and immune function, may increase susceptibility to the harmful effects of air pollution, infection, and inflammation.^30,31^ Moreover, the high prevalence of preexisting conditions among the elderly may further amplify their vulnerability to environmental pollutants.^30,31^

In this study, we assessed the individual effects of six air pollutants and the potential joint effect of air pollution mixtures on 484 plasma proteins in 208 elderly participants from the established and well-characterized Cancer Prevention Study-II (CPS-II) Nutrition Cohort. The objective of this work was to enhance the understanding of key proteins and their biological processes associated with long-term air pollution exposure in this vulnerable population.

## Methods

### Study Design and Population

We included 230 participants with protein profiles measured previously from the CPS-II Nutrition Cohort,^32,33^ a well-established prospective cohort of 184,194 participants with a median age of 63 years residing in 21 states across the US. In 1992–1993, CPS-II Nutrition Cohort participants completed a mailed baseline questionnaire to provide detailed demographic, medical, and behavioral information. Follow-up questionnaires were administered biennially beginning in 1997 to update the information. Between 1998 and 2001, a subcohort of approximately 40,000 participants provided a nonfasting blood sample at community hospitals. These blood samples were shipped overnight with coolant packs to a central repository, where they were aliquoted and frozen at −130 °C for long-term storage. Details of the CPS-II Nutrition Cohort can be found elsewhere.^34^ All aspects of the CPS-II Nutrition Cohort were reviewed and approved by the Emory University Institutional Review Board.

The 230 participants (115 matched lung cancer case–control pairs) in this study were initially included as part of a prior study aimed at identifying protein markers of imminent lung cancer in individuals with a smoking history.^32,33^ The study selected all lung cancer cases diagnosed within three years after blood draw and then matched them individually to controls by age at blood draw, date of blood draw, sex, and race. The comparison of population characteristics between the 230 participants and the entire group of ever-smokers who provided blood samples in the CPS-II Nutrition Cohort can be found in Table S1.

### Retrospective Model-Based Air Pollution Measurement

Ambient air pollution exposure was obtained from the Center for Air, Climate, and Energy Solutions (CACES) database using the residential addresses collected at the time of blood draw at the census block group level. Since our goal was to investigate alterations in plasma protein levels associated with long-term air pollution exposure, we used annual average exposure levels as exposure metrics. Briefly, the CACES database provided annual average exposure levels of six criteria air pollutants for the contiguous US.^35^ The CACES database was constructed using integrated empirical geographic regression models, based on land-use regression models with dimension-reduced predictors using partial least squares from the geographic variables offered. Considering all air pollutants and years, the median *R*-squared (*R* ^2^) based on conventional 10-fold cross-validation (CV) for the models with the best performance was 0.66 (interquartile range (IQR): 0.57–0.83).^35^

We included six air pollutants in our analysis: PM_2.5_, PM_10_, O_3_, NO_2_, SO_2_, and CO. Participants were assigned annual average pollutant levels in the calendar year of the blood draw (between 1998 and 2001). All pollutant data were first available starting in 1999. We assessed the Pearson correlations among the annual average exposure levels for each air pollutant in 1999, 2000, and 2001. The correlation coefficients ranged from 0.84 to 0.97 (Table S2), indicating that air pollution exposure levels were highly correlated across these years. Therefore, we used 1999 measurements as reasonable proxies for 1998 exposures (*N* = 8) to ensure consistency across all pollutants.

### Plasma Proteomic Profiling

The nonfasting plasma samples were utilized for proteomic profiling using the Olink proteomics platform.^36^ This platform, based on proximity extension assays, enables high-throughput, semiquantified measurement of concentrations for highly annotated proteins in less than 50 μL of plasma. Relative concentrations of 552 proteins were measured on six panels: cardiovascular III, inflammation, immuno-oncology, oncology II, oncology III, and neuro Exploratory, through quantitative PCR (qPCR). These proteins are validated biomarkers for cardiovascular studies, inflammatory processes, immune-oncology, oncology, and neurology studies. Cases and controls were randomly assigned to plates, with matched pairs placed on the same plate when applicable. Internal controls were added to each sample to monitor the quality of the assay performance and the quality of individual samples. Samples that did not pass quality control were removed. Details on plasma proteomic profiling can be found elsewhere.^32^

The intra-assay coefficient of variance (%CV) ranged from 3% to 7% (reference intra-assay CV: <15%), while the inter-assay CV ranged from 7% to 11% (reference interassay CV: <25%), indicating high data quality. For proteins measured on multiple panels (with more than one measurement), the measurement with the highest variance was used. Proteins with >20% of values below the limit of detection (LOD) were excluded. However, no proteins met this criterion. For proteins with >80% of detectable values, any values below the LOD were imputed as the LOD divided by the square root of 2. Relative protein concentrations were log_2_-transformed and autoscaled to approximate a normal distribution. Ultimately, 484 distinct proteins were included in the subsequent analyses.

We used the Ensemble Methods for Outlier Detection (EnsMOD) software program to detect potential outliers in protein profiles.^37^ This software incorporates robust strategies, including hierarchical cluster analysis (HCA) using three distance functions combined with five linkage functions, as well as two robust principal component analysis (rPCA) approaches.^38,39^ The software is straightforward to apply and allows for the mutual validation of results. The workflow and parameters are detailed in Supporting Information (see Section Outliers Detection in Protein Profiles). Based on consistent outlier identification by both HCA and the rPCA methods, we removed three samples from the data set.

### Statistical Analysis

We performed a descriptive analysis of covariates, presenting continuous variables as mean and standard deviation and categorical variables as count (*n*) and frequency (%). The characteristics and air pollution exposure levels of former and current smokers were compared using the Wilcoxon rank-sum test for continuous variables and Pearson’s chi-squared test or Fisher’s exact test for categorical variables. We analyzed Pearson correlations between the annual average levels of each pair of air pollutants.

#### Individual Effect and Overall Mixture Effect of Air Pollutants on Plasma Proteins

We identified a list of potential covariates based on a comprehensive literature review and the use of a directed acyclic graph (DAG) (Figure S1). The definitions and collection times of these covariates are detailed in Table S3. To mitigate the risk of compromising statistical power and overadjustment, we applied stepwise selection models to determine the final list of covariates. The covariates retained in the subsequent analyses were age at blood draw (continuous), gender (categorical: male, female), body mass index (BMI) (categorical: <18.5 kg/m^2^, 18.5 to <25 kg/m^2^, 25 to <30 kg/m^2^, ≥30 kg/m^2^), education level (categorical: less than high school, high school graduate, some college or associate’s degree, bachelor’s degree and above), alcohol consumption (categorical: not current drinker, < 1 drink/day, 1 drink/day, 2 or more drinks/day, unknown), fruit and vegetable consumption (categorical: first, second, third, and fourth quartile, unknown), smoking status (categorical: former, current smoker), and pack-years (continuous). The detailed selection process is described in Supporting Information (see Section Stepwise Selection Models to Select the Final List of Covariates).

We applied two approaches to evaluate the associations between long-term exposure to air pollution and plasma proteins. In the first approach, we assessed the effect of individual air pollutants on each plasma protein using multiple linear regression models. The standardized log_2_-transformed concentration of each protein was regressed on the annual average level of each air pollutant, adjusting for the final list of selected covariates. The effect estimates were expressed as changes in the standardized log_2_-transformed concentration of proteins per 1/2 interquartile range (IQR) increase in air pollutant levels, controlling for covariates. Secondarily, we explored the overall effect of the air pollution mixture on each plasma protein using quantile g-computation models. Quantile g-computation provides a single effect estimate for an exposure mixture, offering simplicity in interpretation and computational ease without assuming directional homogeneity.^40^ Additionally, it provides a set of weights that reflect the contribution of each exposure to the overall effect estimate, indicating either a positive or negative partial effect. The standardized log_2_-transformed concentration of each protein was regressed on the annual average level of all air pollutants, adjusting for the final list of selected covariates. For comparison with the individual air pollutant models, the effect estimates were expressed as changes in the standardized log_2_-transformed concentration of proteins per one quartile (25%) increase in all air pollutant levels, controlling for covariates. This analysis was conducted using the “*qgcomp.noboot”* function from the “*qgcomp*” package. The false discovery rate of multiple hypothesis tests was controlled with the Benjamini–Hochberg (BH) procedure. A BH-corrected false discovery rate (FDR) <0.2 was considered statistically significant.

To examine whether cancer status affects the association between air pollution exposure and plasma protein levels, we incorporated cancer status (categorical: case/control) into the models. In addition, we performed separate analyses for the cases and controls. We acknowledge that stepwise selection is not commonly intended for covariate selection. To examine the potential impact of additional covariates, including multivitamin use, passive smoke exposure, time since the last meal, and year of blood draw, we performed a separate set of sensitivity analyses that included these variables in the models.

#### Pathway Enrichment and Protein–Protein Interaction (PPI) Network Analyses

To gain more insights into biological processes and protein–protein interactions related to the air pollution-associated proteins that we identified, we performed pathway enrichment and PPI network analyses. To ensure a sufficient number of proteins in these two analyses, we included the proteins associated with air pollution metrics at unadjusted *p* < 0.05 from both individual air pollutant models and air pollution mixture models. For pathway enrichment analysis, we used the “*enrichKEGG*” function from “*clusterProfiler*” package. The background proteins were customized and consisted of 484 proteins. The function further provides BH-corrected FDR for each pathway. For PPI analysis, we used the Search Tool for the Retrieval of Interacting Genes/Proteins (STRING) database (http://string-db.org/) to identify the interaction relationships. The results were imported into Cytoscape for the visualization of the interaction network.

#### Interaction between Air Pollution Exposure and Smoking

For individual air pollutant models, we further investigated the interaction between long-term air pollution exposure levels and smoking status on plasma proteins. We used the “*visreg*” package to visualize the associations between air pollution exposure levels and standardized log_2_-transformed protein levels by smoking status, holding all other variables constant.

## Results

### Population Characteristics and Air Pollution Exposure Levels

We excluded participants 1) with missing residential history information (*N* = 9); 2) with incomplete covariate information (*N* = 11); and 3) identified as protein profile outliers (*N* = 3). The final sample included 208 participants.

The average age of participants at the time of the blood draw was 70.8 (±5.1) years. All participants were White, and 65% were male (Table 1). Of these participants, 80% were former smokers with an average smoking history of 36.6 (±30.6) pack-years, while 20% were current smokers with an average of 46.9 (±23) pack-years. There were 38% of participants who maintained a healthy weight (18.5 to <25 kg/m^2^), and 38% had attained a bachelor’s degree or higher. Alcohol use and multivitamin use were reported by 69% and 52% of participants, respectively. Significant differences were observed between former and current smokers in terms of age, education levels, pack-years, alcohol use, fruit and vegetable consumption, passive smoke exposure, and year of blood draw.

**Table 1:** Characteristics of the Study Participants with Proteomics Profiling in the Cancer Prevention Study-II Nutrition Cohort (*N* = 208)

	Overall	Former Smokers	Current Smokers	*p*-value^a^
**Variable** ^b^	(*N* = 208)	(*N* = 166)	(*N* = 42)	
Age (years)	70.8 ± 5.1	71.3 ± 4.9	69.2 ± 5.7	0.027
Race				
White	208 (100)	166 (100)	42 (100)	NA
Gender				0.4
Male	136 (65)	111 (67)	25 (60)	
Female	72 (35)	55 (33)	17 (40)	
Lung cancer status				0.5
Control	104 (50)	81 (49)	23 (55)	
Case	104 (50)	85 (51)	19 (45)	
Body mass index				0.2
<18.5 kg/m^2^	2 (1.0)	1 (0.6)	1 (2.4)	
18.5 to <25 kg/m^2^	80 (38)	63 (38)	17 (40)	
25 to <30 kg/m^2^	100 (48)	84 (51)	16 (38)	
≥30 kg/m^2^	26 (13)	18 (11)	8 (19)	
Education level				0.014
Less than high school	17 (8.2)	13 (7.8)	4 (9.5)	
High school graduate	45 (22)	30 (18)	15 (36)	
Some college or associate’s degree	67 (32)	52 (31)	15 (36)	
Bachelor’s degree and above	79 (38)	71 (43)	8 (19)	
Pack-years	38.7 ± 29.5	36.6 ± 30.6	46.9 ± 23.0	0.003
Alcohol consumption				0.002
Not current drinker	58 (28)	41 (25)	17 (40)	
<1 drink/day	73 (35)	66 (40)	7 (17)	
1 drink/day	37 (18)	32 (19)	5 (12)	
2 or more drinks/day	34 (16)	25 (15)	9 (21)	
Unknown	6 (2.9)	2 (1.2)	4 (9.5)	
Multivitamin use				0.4
Not current user	88 (42)	71 (43)	17 (40)	
Current user	109 (52)	88 (53)	21 (50)	
Unknown	11 (5.3)	7 (4.2)	4 (9.5)	
Fruit and vegetable consumption				0.007
First quartile	51 (25)	39 (23)	12 (29)	
Second quartile	47 (23)	38 (23)	9 (21)	
Third quartile	47 (23)	39 (23)	8 (19)	
Fourth quartile	42 (20)	39 (23)	3 (7.1)	
Unknown	21 (10)	11 (6.6%)	10 (24)	
Passive smoke exposure	126 (61)	88 (53)	38 (90)	<0.001
Year of blood draw				0.022
1998	8 (3.8)	6 (3.6)	2 (4.8)	
1999	69 (33)	61 (37)	8 (19)	
2000	121 (58)	94 (57)	27 (64)	
2001	10 (4.8)	5 (3.0)	5 (12)	
Time since last meal				0.2
<2h	109 (52)	90 (54)	19 (45)	
2–4 h	85 (41)	67 (40)	18 (43)	
>4h	12 (5.8)	7 (4.2)	5 (12)	

a The characteristics of former and current smokers were compared using the Wilcoxon rank-sum test for continuous variables and Pearson’s chi-squared test or Fisher’s exact test for categorical variables.

b The continuous variables are presented as mean ± standard deviation, while the categorical variables are presented as count (frequency (%)). The definitions and collection times of these covariates are detailed in Table S3.

The annual mean ± standard deviation concentrations of PM_2.5_, PM_10_, NO_2_, O_3_, SO_2_, and CO in the calendar year of blood draw were 12.9 ± 2.8 μg/m^3^, 21.2 ± 6.0 μg/m^3^, 13.6 ± 6.1 ppb, 48.5 ± 7.0 ppb, 3.5 ± 1.7 ppb, and 0.5 ± 0.2 ppm, respectively (Table 2). The annual average levels of SO_2_ were significantly higher in current smokers compared to those of former smokers, while other pollutant levels were similar across groups (Table 2). Pearson correlations among annual average levels of air pollutants ranged from −0.145 to 0.825. Details can be found in Figure S2.

**Table 2:** Annual Average Exposure Levels of Six Air Pollutants of the Study Population

	Overall	Former Smokers	Current Smokers	
**Air pollution exposure levels** ^a^	(*N* = 208)	(*N* = 166)	(*N* = 42)	***p*** **-value** ^b^
PM_2.5_ (μg/m^3^)	12.9 ± 2.8	12.9 ± 2.9	13.1 ± 2.3	0.2
PM_10_ (μg/m^3^)	21.2 ± 6.0	21.3 ± 6.4	21.2 ± 4.0	0.2
NO_2_ (ppb)	13.6 ± 6.1	13.6 ± 6.5	13.5 ± 4.0	0.4
O_3_ (ppb)	48.5 ± 7.0	48.4 ± 7.2	49.1 ± 5.7	0.4
SO_2_ (ppb)	3.5 ± 1.7	3.4 ± 1.5	4.1 ± 2.1	0.043
CO (ppm)	0.5 ± 0.2	0.5 ± 0.2	0.5 ± 0.1	>0.9

a Annual average air pollution exposure levels in the year of blood draw. PM_2.5_, fine particulate matter; PM_10_, coarse particulate matter; NO_2_, nitrogen dioxide; O_3_, daily 8 h maximum ozone; SO_2_, sulfur dioxide; CO, carbon monoxide.

b The air pollution exposure levels of former and current smokers were compared using the Wilcoxon rank-sum test.

### Individual Air Pollutants and Air Pollution Mixtures Were Associated with Multiple Plasma Proteins, with Some Consistency

We identified associations between multiple plasma proteins and individual air pollutants, as well as air pollution mixtures. Specifically, we observed 23, 48, 47, 46, 45, 36, and 22 proteins associated with PM_2.5_, PM_10_, O_3_, NO_2_, SO_2_, CO, and air pollution mixtures, respectively (unadjusted *p* < 0.05) (Table S4), resulting in a total of 167 distinct proteins. Among these, eight, two, six, and one protein were associated with PM_10_, O_3_, NO_2_, and CO, respectively, meeting the criterion of a BH-corrected false discovery rate (FDR) <0.2 (Table 3). Notable proteins in this category included MCP-4, fibroblast growth factor 5 (FGF-5), CD5, interferon-gamma (IFN-gamma), and IL32. Specifically, per half IQR increase in PM_10_, NO_2_, and CO, the log_2_-transformed concentration of IL32 decreased by 0.097, 0.127, and 0.114 standard deviations, respectively (Table 3). However, no proteins were found to be significantly associated with PM_2.5_, SO_2_, or air pollution mixtures after correction for multiple testing. We found five proteins associated with at least four exposure metrics, either individual air pollutants or a mixture (Figure 1). They were IL32, disintegrin and metalloproteinase domain-containing protein 15 (ADAM15), probable serine carboxypeptidase (CPVL), IL8, and lysosome-associated membrane glycoprotein 3 (LAMP3). Nine ILs and five IL receptors were associated with at least one air pollution metric. Additionally, we observed 15 chemokines associated with at least one air pollution metric.

**Table 3:** Proteins Significantly Associated with Air Pollution Exposure (FDR <0.2)^a^

Protein	Effect Estimate	95% CI	*p*	FDR	Associated Pollutant
MCP-4	–0.121	(−0.184, −0.059)	0.000	0.088	PM_10_
FGF-5	–0.108	(−0.176, −0.040)	0.002	0.139	PM_10_
CD5	–0.096	(−0.154, −0.038)	0.001	0.139	PM_10_
IFN-gamma	–0.103	(−0.168, −0.039)	0.002	0.139	PM_10_
IL32	–0.097	(−0.157, −0.037)	0.002	0.139	PM_10_
CRNN	–0.107	(−0.174, −0.040)	0.002	0.139	PM_10_
HBQ1	–0.098	(−0.160, −0.037)	0.002	0.139	PM_10_
IL2	–0.096	(−0.160, −0.033)	0.003	0.195	PM_10_
IL32	–0.127	(−0.193, −0.061)	0.000	0.091	NO_2_
CX3CL1	–0.118	(−0.185, −0.052)	0.001	0.134	NO_2_
IGFBP-2	0.102	(0.038, 0.166)	0.002	0.179	O_3_
CXCL10	0.109	(0.040, 0.179)	0.002	0.179	O_3_
LAMP3	0.098	(0.036, 0.161)	0.002	0.179	O_3_
MMP12	0.102	(0.041, 0.164)	0.001	0.179	O_3_
CD83	0.110	(0.041, 0.179)	0.002	0.179	O_3_
TNFRSF4	0.107	(0.042, 0.172)	0.001	0.179	O_3_
IL32	–0.114	(−0.176, −0.052)	0.000	0.196	CO

a Note: The standardized log_2_-transformed concentration of each protein was regressed on the annual average level of each air pollutant, controlling for age at blood draw, gender, BMI, education level, alcohol use, fruit and vegetable consumption, smoking status, and pack-years. The effect estimates were expressed as changes in the standardized log_2_-transformed concentration of proteins per 1/2 interquartile range increase in air pollutant levels, controlling for covariates. CI, confidence interval; FDR, Benjamini–Hochberg corrected false discovery rate; PM_10_, coarse particulate matter; NO_2_, nitrogen dioxide; O_3_, daily 8 h maximum ozone; CO, carbon monoxide.

**Figure 1:**
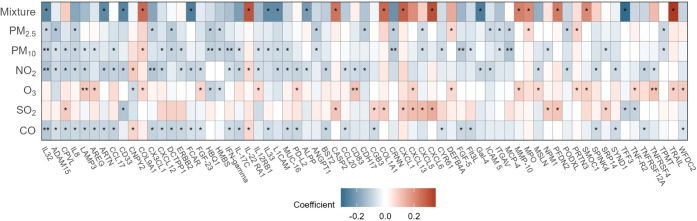
Heatmap of air pollutant-protein associations (unadjusted *p* < 0.05 or BH-corrected FDR < 0.2). Each rectangle represents one air pollutant-protein association. The *y*-axis listed each air pollutant or air pollution mixture, while the *x*-axis listed each protein. From left to right, the proteins are ordered by the number of air pollutant-protein associations at unadjusted *p* < 0.05. The color indicates the effect coefficient. For individual air pollutant effects, the effect estimates were expressed as changes in standardized log_2_-transformed levels of proteins per1/2 interquartile range increase in air pollutant levels, controlling for covariates. For overall mixture effects, the effect estimates were expressed as changes in standardized log_2_-transformed levels of proteins per one quartile (25%) increase in all air pollutant levels, controlling for covariates. The asterisk indicates the significance level: *, unadjusted *p* < 0.05; **, BH-corrected FDR < 0.2. Note: PM_2.5_, fine particulate matter; PM_10_, coarse particulate matter; NO_2_, nitrogen dioxide; O_3_, daily 8 h maximum ozone; SO_2_, sulfur dioxide; CO, carbon monoxide; Mixture, air pollution mixture.

Twenty proteins were identified as being associated with both air pollution mixtures and at least one individual air pollutant (unadjusted *p* < 0.05), including IL32 and artemin (ARTN) (Figure 1). Overall, the effect estimates for proteins associated with air pollution from the mixture model were larger than those derived from the individual pollutant models. Additionally, the direction of effect estimates for overlapping proteins was consistent across both approaches. Weights corresponding to the partial effect of individual air pollutants for significant proteins in air pollution mixture-protein models (unadjusted *p* < 0.05) are presented in Figure S3. We observed fewer proteins associated with the air pollution mixture model compared with individual air pollutant models (Table S4). All results from the air pollution-protein analyses can be found in Tables S1–S7.

Including the cancer status in the models has a minimal impact on the results. Among proteins identified at unadjusted *p* < 0.05, we observed 74% to 100% overlap in signals across all air pollution metrics after adjusting for cancer status (Table S8). The number of proteins associated with any air pollution metrics at FDR <0.2 decreased from 15 to 3 distinct proteins (Table S9). When performing the analyses among cases and controls separately, we found the main results were primarily driven by controls (*N* = 104), as evidenced by a greater overlap in signals between the overall study population and controls (Tables S10 and S11). The changes in results were largely attributed to the significant reduction in the sample size. To increase the statistical power, we retained lung cancer cases in the analysis. After adjustment for additional covariates, the results remained largely consistent. We observed a 51% to 94% overlap in signals across all air pollution metrics at unadjusted *p* < 0.05 after additional adjustment (Table S12). The number of proteins identified at FDR <0.2 decreased from 15 to 7 distinct proteins (Table S13). To minimize overadjustment and increase statistical power, we presented results from reduced models as the main results.

### Air Pollution Exposure Was Associated with Biological Pathways Related to the Immune System and Signaling

We did not observe any pathways meeting BH-corrected FDR <0.2. Therefore, pathways with unadjusted *p* < 0.05 were presented. We identified two, one, one, and one pathway associated with PM_10_, NO_2_, O_3_, and CO (Figure 2, Table S14), respectively, which were the PI3K-Akt signaling pathway, ErbB signaling pathway, cytokine–cytokine receptor interaction, chemokine signaling pathway, and cell adhesion. We did not observe any pathways associated with PM_2.5_, SO_2_, or the air pollution mixture.

**Figure 2:**
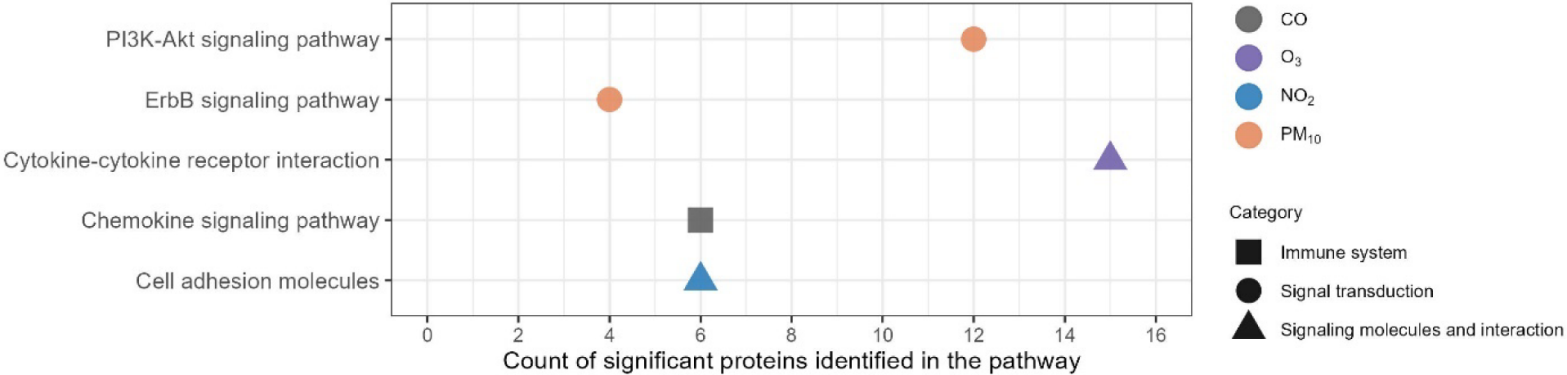
Biological pathways associated with air pollution exposure (unadjusted *p* < 0.05). The proteins of interest were those associated with air pollution at unadjusted *p* < 0.05 from individual air pollutant models and air pollution mixture models. We observed no pathways associated with PM_2.5_, SO_2_, and an air pollution mixture. Note: PM_2.5_, fine particulate matter; PM_10_, coarse particulate matter; NO_2_, nitrogen dioxide; O_3_, daily 8 h maximum ozone; SO_2_, sulfur dioxide; CO, carbon monoxide.

The interaction networks for proteins associated with air pollution mixtures are displayed in Figure 3, while those for individual air pollutants are displayed in Figure S4. Most proteins were positively associated with O_3_, SO_2_, and an air pollution mixture, but negatively associated with PM_2.5_, PM_10_, NO_2_, and CO.

**Figure 3:**
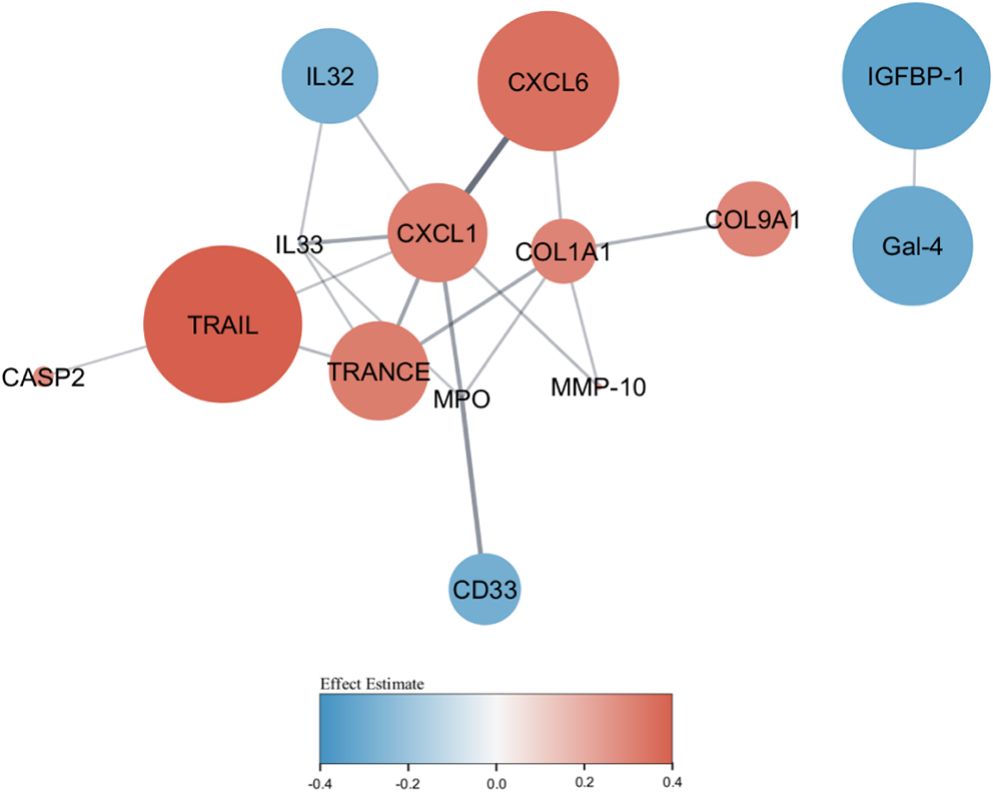
Protein–protein interaction network of air pollution mixture-associated proteins. The proteins of interest were those associated with air pollution at unadjusted *p* < 0.05 from air pollution mixture models. Larger circles represent smaller *p-*values. The red color represents a positive association between air pollution mixtures and the processed level of protein, while blue represents a negative association between air pollution mixtures and the processed level of protein. The darker the color, the larger the effect estimates. The lines represent the connections between proteins. The thicker the line, the stronger the evidence supporting that connection. The effect estimates were expressed as changes in standardized log_2_-transformed levels of proteins per one quartile (25%) increase in all air pollutant levels, controlling for covariates.

### The Associations of Air Pollution Exposure with Proteins Varied among Current and Former Smokers

No significant interaction term was observed at FDR < 0.2. However, we found that the association of air pollution exposure with 99 distinct proteins varied by smoking status when using a loosened statistical threshold (*p*-value for the interaction term <0.05). There were six, 15, 18, 42, 36, and 16 interaction terms at *p*-value <0.05 for PM_2.5_, PM_10_, NO_2_, O_3_, SO_2_, and CO, respectively. For example, among individuals exposed to equivalent levels of PM_2.5_, PM_10_, NO_2_, O_3_, and CO, current smokers exhibited a greater reduction in the standardized log_2_-transformed concentration of IL8, a key mediator of the inflammatory response, compared to former smokers. Additionally, the effects of PM_10_, NO_2_, SO_2_, and CO on group 10 secretory phospholipase A2 (PLA2G10) were attenuated in current smokers compared to former smokers. PLA2G10 is a critical enzyme involved in the generation of inflammatory lipid mediators. Overall, the modification effects of smoking status on the relationship between air pollution and protein levels varied across different air pollutants and proteins. Notably, the effect of O_3_ on elevated protein levels was attenuated across 42 proteins in current smokers compared to those in former smokers. Among the 99 distinct proteins for which air pollutants interacted with smoking status, the main effects of air pollutants and smoking status were also significant for 25 distinct proteins, with unadjusted *p*-value <0.05. Only proteins where the effect estimates of air pollutants, smoking status, and the interaction term in individual air pollutant-protein models were at *p* < 0.05 are plotted and displayed in Figure S5. The summary of proteins in different categories is presented in Supporting Information. Detailed model parameters of proteins with interaction terms at unadjusted *p* < 0.05 can be found in Table S15.

## Discussion

In this study, we evaluated the individual and potential joint effects of six ubiquitous air pollutants on 484 plasma proteins in 208 elderly participants from the CPS-II Nutrition Cohort. We identified numerous proteins significantly associated with various individual air pollutants and air pollution mixtures, primarily those involved in immune responses, inflammation, cell signaling, and cell growth. Twenty proteins (FDR <0.2 or *p* < 0.05) were associated with both air pollution mixtures and at least one individual air pollutant, with consistent directions of association across pollutant metrics. We also found that the effects of air pollution exposures on several plasma proteins differed by smoking status (former vs current). Overall, the findings offer valuable insight into the chronic biological response in plasma protein levels to air pollution exposure.

A key finding from the current analyses was the identification of 167 distinct proteins associated with either air pollution mixtures or individual air pollutants (*p* < 0.05). In particular, five of these proteins were associated with ≥4 exposure metrics, and 15 met the FDR < 0.2 threshold. Nine ILs and five IL receptors were associated with at least one air pollution metric. IL32, IL8, IL-17C, IL33, and IL2 were negatively associated , while IL1 alpha and beta, IL4, and IL17F were positively associated with air pollution exposure. Specifically, IL32 stimulates the production of multiple cytokines, including TNF-alpha, IL8, and IFN-gamma, and activates several cytokine signaling pathways, potentially playing a crucial role in both innate and adaptive immune responses.^41^ Due to its diverse functions in inflammatory conditions, IL32 has been associated with several diseases, including chronic obstructive pulmonary disease (COPD),^42^ asthma,^43^ type 2 diabetes,^44^ and multiple cancers,^45^ and has been suggested as a potential biomarker and therapeutic target.^43,46,47^ Consistently, we found that IL32 had robust and consistent negative associations with PM_10_, NO_2_, and CO (FDR < 0.2), as well as with PM_2.5_ and air pollution mixtures (*p* < 0.05). Additionally, IL8 was inversely associated with PM_2.5_, PM_10_, NO_2_, and CO (*p* < 0.05), and IL2 was inversely associated with PM_10_ (FDR < 0.2). Previous studies have reported inverse associations between IL8 and PM_10_ exposure in young girls, as well as between IL8 and IL2 with NO_*x*_ exposure in adults.^20,29^ However, Mostafavi et al. did not observe significant associations of IL1 beta, IL2, and IL4 with NO_*x*_ in adults.^29^ In contrast to these previous findings, we found that IFN-gamma, a key cytokine stimulating macrophages and involved in antitumor and antiviral immunity, was negatively associated with PM_10_ (FDR < 0.2), NO_2_, and CO exposure (*p* < 0.05).^20^ In our study, IL6, IL10, and TNF-alpha were not associated with air pollution exposure. Previous epidemiological studies have shown their associations with air pollution exposure, but the findings were inconsistent.^18,29,48,49^

It is worth noting that we observed 15 chemokines associated with at least one air pollution metric. In particular, MCP-4 (CCL13) was inversely associated with PM_10_ (FDR < 0.2) and PM_2.5_ (*p* < 0.05), consistent with the direction of effects reported by Rothman et al.^50^ MCP-4 plays an important role in recruiting leukocytes during allergic and nonallergic inflammation.^51^ In addition, previous studies have reported negative associations between air pollution exposure and CCL17 and CXCL8 ^52^ and positive associations between PM components and CXCL1, CXCL5, and CXCL10,^53^ which align with our findings. However, they also observed positive associations between air pollution exposure and CXCL1 and CCL11,^52^ as well as negative associations with CXCL8, CXCL11, and CCL20, which contrast with our observations.^53^

We found that higher air pollution exposure was negatively associated with multiple proteins such as ADAM15, FGF-5, and CD5. ADAM15 is a key protein in the ADAM family. ADAMs are multidomain transmembrane proteins that are critical in proteolysis, cell adhesion, and cell migration, which are essential for immune regulation and inflammation.^54,55^ ADAM15 has been implicated in the development of several diseases, such as cancer, Alzheimer’s disease, and chronic immune disorders.^54,56,57^ FGF-5 plays a vital role in cell proliferation and cell differentiation and has been implicated in cancer formation and tissue regeneration.^58–60^ CD5 is important for lymphocyte selection and immune tolerance. In summary, the downregulation of these proteins may imply perturbation of cellular growth and interaction, as well as immune and inflammatory responses. The negative associations between air pollution and these proteins may suggest perturbation in cellular growth and interactions, as well as alterations in immune and inflammatory responses.^61^ Taken together, numerous plasma proteins, primarily those involved in immune responses, inflammation, cell signaling, and cell growth, were found to be associated with either air pollution mixtures or individual air pollutants. Aside from those previously linked to air pollution exposure, multiple newly identified proteins, such as IL32, ADAM15, and MCP-4, warrant further investigation. Long-term exposure to environmental pollutants, such as air pollution, can cause immune disorders due to their continuous interaction with immune responses.^62^ Persistent oxidative stress and inflammation caused by chronic air pollution exposure may impair immune cell function, reduce production, or increase depletion of certain cytokines, ultimately weakening immune responses and altering immune-related protein profiles. Therefore, this may potentially explain the observed negative associations between long-term exposure to air pollution and reduced levels of multiple immune-related proteins, such as IL32, IL8, and ADAM15, which are indicative of potential immune disorders.

We observed five biological pathways in which air pollution-associated proteins were enriched, including the PI3K-Akt signaling pathway, ErbB signaling pathway, cytokine–cytokine receptor interaction, chemokine signaling pathway, and cell adhesion molecules. The ErbB signaling pathway and its downstream PI3K-Akt signaling pathway play critical roles in cell survival, growth, and proliferation, which can be activated by cytokines and chemokines.^63,64^ Studies in mice and cellular models have demonstrated that PM_2.5_ exposure may induce autophagy-mediated pulmonary cell apoptosis via activation of the oxidative-stress PI3K-Akt signaling pathway.^65,66^ The PI3K-Akt signaling pathway has been associated with various diseases, including respiratory conditions such as COPD,^67^ cancers,^68^ cardiovascular diseases,^69^ neurological disorders,^70^ and inflammatory diseases.^71^ Moreover, exposure to PM_2.5_ has been linked to the dysregulation of the ErbB family of receptors,^72^ which can promote oncogenesis.^73,74^ The cytokine–cytokine receptor interaction and chemokine signaling pathway regulate cell migration, immune responses, and inflammation, frequently involving the activation of the PI3K-Akt signaling pathway.^75–77^ Additionally, cell adhesion molecules are influenced by both cytokines and chemokine signaling, as well as by the activation of the PI3K-Akt and ErbB signaling pathways, which are critical in immune cell trafficking and cancer metastasis.^78^ Air pollution exposure has been associated with cytokine–cytokine receptor interaction,^79^ the expression of chemokines,^52^ and cell adhesion.^80,81^ In conclusion, these pathways are tightly interconnected and closely linked to immune responses, inflammation, cellular growth, and cell migration, which may mediate the adverse effects of air pollution exposure. We observed no pathways associated with PM_2.5_, SO_2_, and an air pollution mixture, which may be due to several factors. First, the study may lack sufficient statistical power to detect more air pollution-associated proteins due to the limited sample size. Second, variability in sources, chemical composition, and temporal and spatial patterns of air pollutants—particularly PM, which is a complex mixture—were not accounted for in the protein analyses. These factors are essential considerations in assessing the toxicity of air pollutants, particularly in this study, which includes participants from various regions across the US.^82–84^

Using quantile g-computation models, we identified fewer proteins associated with the air pollution mixture compared with individual pollutant models analyzed via linear regression, contrary to our expectations. As previously suggested, we hypothesized that potential additive or synergistic effects among air pollutants could lead to more extensive protein perturbation.^85^ One possible explanation is that the presence of heterogeneous effects among air pollutants may lead to fewer significant associations. Additionally, uncertainties in exposure characterization, arising from the varying performance of prediction models for different air pollutants,^35^ could introduce and amplify uncertainties in estimating the joint effects of the entire mixture. Twenty proteins were found to be associated with both air pollution mixtures and at least one individual pollutant, with consistent directions of association across both approaches. Notably, the effect estimates for proteins with air pollution from the mixture model were, in general, larger than those derived from individual pollutant models, aligning with our expectations. In conclusion, our findings indicated that while individual pollutant models may serve as reasonable surrogates for air pollution mixture exposure, they may underestimate the effects of air pollution, potentially biasing the results toward the null.

More interestingly, our findings also revealed that the impact of air pollution exposure on protein levels may differ based on current or former smoking status. Air pollution and cigarette smoke are both inhaled through the respiratory tract, inducing local and systemic oxidative stress and inflammation.^86,87^ However, research on their combined effects remains limited and shows inconsistency. A previous meta-analysis indicated that associations between PM_2.5_ and lung cancer risk were stronger in former smokers, followed by never-smokers, and then current smokers.^88^ However, two other studies, including the one performed in the CPS-II cohort, found that the effects of air pollution exposure, mainly PM, on reduced pulmonary function and increased lung cancer mortality were more pronounced among current smokers compared to never-smokers.^89,90^ In our current analysis, we observed that the modification effects of smoking status on the relationship between air pollution and protein levels varied across different air pollutants and proteins. Specifically, the effects of each air pollutant, including PM_2.5_ and PM_10_, on the majority of the proteins with significant interaction effects, were attenuated in current smokers compared to former smokers. A previous study observed that healthy smokers exhibited a less pronounced decline in lung function and fewer symptoms in response to O_3_ exposure compared to nonsmokers.^91^ Consistently, in the current study, the effect of O_3_ on elevated protein levels was attenuated in current smokers compared to former smokers. To better understand the potential joint effects and inform intervention, future large-scale investigations are needed on the modification of air pollution impacts by smoking status. A limitation of the current stratified analysis is the small sample size, especially among current smokers, which may result in insufficient statistical power.

This study has several strengths. First, it is based on a well-established prospective cohort with a comprehensive information collection. Second, we considered the potential joint effects of an air pollution mixture on plasma proteins. Third, our study included a large panel of proteins that are validated biomarkers for cardiovascular diseases, inflammatory processes, immune-oncology, oncology, and neurology, providing specific insights into relevant health outcomes and offering a broader scope than previous studies.^21–29^ However, our study also has some limitations. First, the cross-sectional study design prevented us from drawing any causal inference conclusions between air pollution exposure and plasma protein levels. There may be residual confounding from variables such as the season of biosampling and indoor exposures. Although adjusting for cancer status had a minimal impact on the associations of interest, future studies should aim to validate our findings in larger, healthy populations. Second, we did not consider variability in the sources, chemical composition, and temporal and spatial patterns of air pollutants of interest, which are critical factors influencing their toxicity. Although we used validated spatiotemporal models to estimate participants’ exposure levels, we did not have data on individual daily activities or indoor exposures. This may lead to nondifferential exposure misclassification, potentially biasing the estimated effects of air pollution. It is important to note that O_3_ levels peak during the summer due to photochemical formation. Thus, using annual averages may underestimate true exposure and associated health effects by incorporating lower levels in winter. Third, given the exploratory and hypothesis-generating nature of our study, applying a stringent threshold (FDR <0.2) may help limit false positives but also risks missing true associations. Consistent with other exploratory environmental omics investigations,^92–94^ we focused on characterizing proteins associated with each air pollutant or air pollution mixture at an unadjusted *p*-value <0.05 for pathway enrichment and PPI analyses, which may increase the risk of false discoveries. Our results should be validated by future hypothesis-testing studies with larger sample sizes. Fourth, while the application of quantile g-computation to model the joint effects of air pollutants is a novel strength of this study, the relative weights produced by this method are fixed, descriptive, and do not have associated measures of uncertainty. Given their sensitivity to model assumptions and data structure, these weights should be interpreted cautiously and not used as standalone evidence of individual pollutant importance. Lastly, there were significant differences in several characteristics, such as age at blood draw, race, gender, smoking status, and education level, which suggest our sample may not fully represent the entire ever-smokers group in the CPS-II Nutrition Cohort who provided blood samples. Additionally, our study population primarily consisted of elderly White males with a smoking history. Caution should be taken when extrapolating the results to other populations.

## Supplementary Material





## Notes

Table S1 for the comparison of population characteristics between the study sample (*N* = 230) and the entire ever-smokers who provided blood samples in the CPS-II Nutrition Cohort (*N* = 20,822); Table S2 for Pearson correlations among annual average air pollutant exposure levels of each air pollutant across 1999, 2000, and 2001. Table S3 for potential covariates that were considered in statistical analyses; Table S4 for the number of proteins associated with individual air pollutants or the air pollution mixture exposure levels at different thresholds; Table S5 for summary of the interaction between each air pollutant and smoking status (former/current); Figure S1 for a directed acyclic graph (DAG) of relationships among air pollution exposure, plasma protein levels, and covariates; Figure S2 for Pearson correlations among annual average levels of six air pollutants; Figure S3 for weights of individual air pollutants for proteins identified in air pollution mixture-protein models (unadjusted *p* < 0.05); Figure S4 for protein–protein interaction network of individual air pollutant-associated proteins; Figure S5 for the plots for the effects of air pollutants on proteins by smoking status, holding all other variables constant (PDF)
Table S1 for associations between annual average fine particulate matter (PM_2.5_) exposure level and plasma levels of each protein; Table S2 for associations between annual average coarse particulate matter (PM_10_) exposure level and plasma levels of each protein; Table S3 for associations between annual average nitrogen dioxide (NO_2_) exposure level and plasma levels of each protein; Table S4 for associations between annual average daily 8 h maximum ozone (O_3_) exposure level and plasma levels of each protein; Table S5 for associations between annual average sulfur dioxide (SO_2_) exposure level and plasma levels of each protein; Table S6 for associations between annual average carbon monoxide (CO) exposure level and plasma levels of each protein; Table S7 for associations between annual average air pollution mixture exposure and plasma levels of each protein; Table S8 for proteins associated with any air pollution metrics at unadjusted *p* < 0.05 after further including cancer status into the main model; Table S9 for proteins associated with any air pollution metrics at FDR <0.2 after further including cancer status into the main model; Table S10 for proteins associated with any air pollution metrics at unadjusted *p* < 0.05 among controls; Table S11 for proteins associated with any air pollution metrics at FDR <0.2 among controls; Table S12 for proteins associated with any air pollution metrics at unadjusted *p* < 0.05 after further including multivitamin use, passive smoke exposure, time since last meal, and year of blood draw; Table S13 for proteins associated with any air pollution metrics at FDR <0.2 after further including multivitamin use, passive smoke exposure, time since last meal, and year of blood draw; Table S14 for biological pathways associated with air pollution exposure (unadjusted *p* < 0.05); Table S15 for summary of proteins where the effect estimate of the interaction term between air pollutant exposure levels and smoking status in individual air pollutant-protein models was significant (unadjusted *p* < 0.05)­(XLSX)
