# Supplementary material for: The Associations of Air Pollution Mixture Exposure with Plasma Proteins in an Elderly U.S. Panel
Source: Environ Sci Technol. 2025 Jul 24;59(30):15692–704. doi: 10.1021/acs.est.5c03052 (PMC12329713; doi:10.1021/acs.est.5c03052)
Supplement: Supplementary file 1 [file es5c03052_si_001.pdf]

Supplementary materials

The associations of air pollution mixture exposure with plasma proteins in an elderly U.S. panel

Ziyin Tang[1], Ying Wang[2,*], Jeremy A. Sarnat[1], W.Ryan Diver[2,4,5], Todd M. Everson[1], Emily Deubler[2], Youran Tan[1], Stephanie M. Eick[1], Aparna H. Kesarwala[3], Michelle C. Turner[4,5], Carmen J. Marsit[1], Mattias Johansson[6], Hilary A. Robbins[6], Donghai Liang[1,*]

[1]Gangarosa Department of Environmental Health, Rollins School of Public Health, Emory University, Atlanta, Georgia, United States, 30322

[2]Department of Population Science, American Cancer Society, Atlanta, Georgia, United States, 30303

[3]Department of Radiation Oncology, Emory University School of Medicine, Atlanta, Georgia, United States, 30322

[4]Barcelona Institute for Global Health (ISGlobal), Barcelona, Spain, 08036

[5]Universitat Pompeu Fabra (UPF), Barcelona, Spain, 08018

[6]Genomic Epidemiology Branch, International Agency for Research on Cancer, Lyon, France, 69366

***Corresponding authors:**

Donghai Liang, PhD

Associate Professor, Gangarosa Department of Environmental Health

Rollins School of Public Health, Emory University

1518 Clifton Rd NE

Atlanta, GA, 30322

donghai.liang@emory.edu

Ying Wang, PhD

Senior Principal Scientist, Department of Population Science

American Cancer Society, Inc.

270 Peachtree Street NW Suite 1300

Atlanta, GA 30303

ying.wang@cancer.org

Declaration of competing financial interests

The authors declare they have nothing to disclose.

Acknowledgement

The authors sincerely appreciate all CPS-II participants and all members of the study and biospecimen management group. The authors would like to acknowledge the contribution to this study from central cancer registries supported through the Centers for Disease Control and Prevention's National Program of Cancer Registries and cancer registries supported by the National Cancer Institute's Surveillance Epidemiology and End Results Program. We also appreciate members of the Environmental Metabolomics and Exposomics Research Group at Emory (EMERGE) for their valuable input and feedback on this project.

Funding

The American Cancer Society funds the creation, maintenance, and updating of the Cancer Prevention Study-II cohort. Support of this project is from Michel & Claire Gudefin Family Foundation Inc. ZT is supported by the National Cancer Institute (NCI) F99/K00 Award

[F99CA294242]. We also acknowledge the support from the National Institute of Health (NIH) research grants [R21ES032117, R01ES035738] and the HERCULES Exposome Research Center, supported by the National Institute of Environmental Health Sciences of the NIH (P30ES019776). MCT is funded by a Ramón y Cajal fellowship (RYC-2017-01892) from the Spanish Ministry of Science, Innovation and Universities and co-funded by the European Social Fund. ISGlobal acknowledges support from the grant CEX2023-0001290-S funded by MCIN/AEI/ 10.13039/501100011033, and support from the Generalitat de Catalunya through the CERCA Program.

Disclaimer

Where authors are identified as personnel of the American Cancer Society and International Agency for Research on Cancer / World Health Organization, the authors alone are responsible for the views expressed in this article and they do not necessarily represent the decisions, policy or views of the American Cancer Society or the American Cancer Society – Cancer Action Network or International Agency for Research on Cancer / World Health Organization.

Number of pages	29
Number of figures	5
Number of tables	5

Outliers detection in protein profiles

The Ensemble Methods for Outlier Detection (EnsMOD) software program incorporated different methods to complement to each other in identifying potential outliers in omics data.[1]This software incorporates robust strategies mainly hierarchical cluster analyses and robust principal component analyses (rPCA), as demonstrated in two recent publications. [2, 3] The software's performance was tested using anthrax phosphoproteomics data from 46 mouse samples, successfully identifying one deliberately introduced sample outlier. Additionally, studies by Manes et al. and Chen et al. demonstrated that the detection and removal of outliers are crucial for improving the discovery of differentially abundant analytes in omics datasets. [1, 2]

First, we tested the normality of the variation of the protein data. The imputed raw relative abundance of proteins was $\log_2$-transformed and auto-scaled to approximate the normal distribution. The input consisted of processed levels of 484 unique proteins from 230 samples. The density plot of the distribution of processed protein levels indicated similar expression patterns across the samples. The coefficient of determination (R^2) using the empirical density curve of modified z-scores and the standard normal distribution (mean = 0, standard deviation = 1) was 0.997, suggesting the variance closely follows a normal distribution.

Second, hierarchical clustering analyses (HCA) were conducted to assess the proximity of samples to each other. Three distance functions (*Euclidean, Manhattan, and Pearson*) and five linkage functions (*average, Ward.D2, complete, single,* and *centroid*) were used. Among 15 HCAs, the maximum Cophenetic Correlation Coefficient (CCC), calculated using Euclidean distance and centroid linkage, was 0.837 (> 0.800), suggesting that the clustering was significant, and outliers could be detected if any existed. The Silhouette coefficient (SC), an indicator of how strongly each sample belongs to its cluster, was calculated for each sample. A sample with SC < 0.25 is

considered a potential outlier due to poor clustering with other samples. We observed a total of six samples with SC < 0.25.

Lastly, two robust principal component analyses (rPCA) strategies were employed: Robust Sparse PCA algorithm (robpca) [3, 4] and Robust PCA based on Projection Pursuit using GRID (PcaGrid) [2]. These methods identified outliers exceeding a probability threshold, with cutoffs for both robpca and PcaGrid set at 0.975. A total of 24 samples were implied as outliers.

As suggested by Manes et al., [1] using all three criteria is a reasonable strategy for outlier detection. Consequently, we decided to remove three samples identified as outliers by both HCA and the two rPCA strategies.

Stepwise selection models to select the final list of covariates

We conducted stepwise selection models using the '*stepAIC()*' function from '*MASS*' package. This process iteratively adds and removes variables from regression models to identify a set of variables that construct the model with the lowest Akaike information criterion (AIC) value. The final model thus explains the most variations in the data with a parsimonious set of variables.

We fitted multiple linear regressions, using the standardized $\log_2$-transformed concentration of each protein as the dependent variable and the level of each air pollutant as the independent variable. Initial models included all potential covariates, including age at blood draw, gender, BMI, education level, fruit and vegetable consumption, alcohol use, multivitamin use, smoking status, pack-years, passive smoke exposure, year of blood draw, and hours since last meal. Through the iteration process, we set that air pollutant variables were always included in the final models. For each air pollutant-protein association, a single final model was determined.

We extracted the covariates from all final models and calculated the frequency of each variable's occurrence. Variables present in more than 15% of final models were selected as the final covariates.

Table S1. The comparison of population characteristics between the study sample (N=230) and the entire ever-smokers who provided blood samples in CPS-II Nutrition Cohort (N=20,822).

**Variable** [a]	**Study Participants** (N = 230)	**Ever-smokers who provided a blood sample in CPS-II Nutrition Cohort** (N = 20,822)	***P*-value** [b]
**Age at blood draw**	70.9± 5.1	69.6 ± 5.8	<0.001
**Race**			0.036
White	230 (100)	20,465 (98)	
non-White	0 (0)	357 (1.7)	
**Gender**			0.003
Male	146 (63)	11,162 (54)	
Female	84 (37)	9,660 (46)	
**Body mass index**			0.5
< 18.5 kg/m^2	5 (2.2)	617 (3.0)	
18 to <25 kg/m^2	87 (38)	8,323 (40)	
25 to < 30 kg/m^2	104 (45)	8,433 (41)	
≥30 kg/m^2	32 (14)	3,329 (16)	
Unknown	2 (0.9)	120 (0.6)	
**Education level**			0.001
Less than high school	19 (8.3)	769 (3.7)	
High school graduate	54 (23)	4,097 (20)	
Some college/associate degree	70 (30)	6,237 (30)	

Bacehlor's degree and above	85 (37)	9,606 (46)	
Unknown	2 (0.9)	113 (0.5)	
**Smoking status**			<0.001
Former	188 (82)	19,516 (94)	
Current	42 (18)	1,306 (6.3)	
**Alcohol consumption**			0.3
Not current drinker	58 (25)	6,095 (29)	
<1 drink/day	80 (35)	7,496 (36)	
1 drink/day	40 (17)	3,392 (16)	
2 or more drinks/day	38 (17)	2,992 (14)	
Unknown	14 (6.1)	847 (4.1)	
**Multivitamin use**			0.1
Not current user	81 (35)	7,714 (37)	
Current user	127 (55)	11,834 (57)	
Unknown	22 (9.6)	1,274 (6.1)	
**Year of blood draw**			0.2
1998	10 (4.3)	1,445 (6.9)	
1999	76 (33)	5,788 (28)	
2000	132 (57)	12,172 (58)	
2001	12 (5.2)	1,417 (6.8)	
**Fruit and vegetable consumption**			0.052
First quantile	53 (23)	5,191 (25)	
Second quantile	52 (23)	5,003 (24)	
Third quantile	52 (23)	4,886 (23)	
Fourth quantile	52 (23)	4,742 (23)	

Unknown	21 (9.1)	1,000 (4.8)	
**Passive smoke exposure**			0.1
No	50 (22)	5,152 (25)	
Yes	172 (75)	15,304 (73)	
Unknown	8 (3.5)	366 (1.8)	
**Time since last meal**			0.4
<2 hours	120 (52)	11,788 (57)	
2-4 hours	93 (40)	7,651 (37)	
4+ Hours	17 (7.4)	1,378 (6.6)	
Unknown	0	5	

[a]The continuous variables are presented as mean ± standard deviation, while the categorical variables are presented as count (frequency (%)). The definitions and collection time of these covariates are detailed in Table S3. [b] The characteristics between the study sample and the entire ever-smokers who provided blood samples in CPS-II Nutrition Cohort were compared using the Wilcoxon rank sum test for continuous variables and Pearson's Chi-squared test or Fisher's exact test for categorical variables.

Table S2. Pearson correlations among annual average air pollutant exposure levels of each air pollutant across 1999, 2000, and 2001.

$PM_{2.5}$	1999	2000	2001
1999	1.000	0.899	0.879
2000	0.899	1.000	0.945
2001	0.879	0.945	1.000

(A)

PM_{10}	1999	2000	2001
1999	1.000	0.925	0.947
2000	0.925	1.000	0.943
2001	0.947	0.943	1.000

(B)

NO_2	1999	2000	2001
1999	1.000	0.972	0.958
2000	0.972	1.000	0.965
2001	0.958	0.965	1.000

(C)

O_3	1999	2000	2001

1999	1.000	0.856	0.930
2000	0.856	1.000	0.841
2001	0.930	0.841	1.000

(D)

SO_2	1999	2000	2001
1999	1.000	0.943	0.922
2000	0.943	1.000	0.926
2001	0.922	0.926	1.000

(E)

CO	1999	2000	2001
1999	1.000	0.907	0.898
2000	0.907	1.000	0.958
2001	0.898	0.958	1.000

(F)

Note: $PM_{2.5}$, fine particulate matter; PM_{10}, coarse particulate matter; NO_2, nitrogen dioxide; O_3, daily 8h maximum ozone; SO_2, sulfur dioxide; CO, carbon monoxide.

Table S3. Potential covariates that were considered in statistical analyses.

**Covariates**	**Definition**	**Time of collection**
Age at blood draw (years)	Continuous	Survey at blood draw
Gender	Categorical: male, female	1982 survey
Body max index (BMI)	Categorical: underweight: $< 18.5\ \mathrm{kg/m^2}$, healthy weight: $18.5 - 25\ \mathrm{kg/m^2}$, overweight: $25 - 30\ \mathrm{kg/m^2}$, obesity: $\geq 30\ \mathrm{kg/m^2}$	Survey at blood draw or 1999 survey
Education level	The highest level of education<br>Categorial: less than high school, high school graduate, some college or associate's degree, bachelor's degree and above	1982 survey
Alcohol use	Categorical: not current drinker, $<$ 1drink/day, $>$ 1drink/day, 2 or more drinks/day, unknown	Survey prior to blood draw (1997 or 1999)
Multivitamin use	Categorical: not current user, current user, unknown	Survey prior to blood draw (1997 or 1999)
Fruit and vegetable consumption	The original variable is continuous in units of servings/day. We categorized it into quartiles based on the distribution of study participants.	1999 survey

	Categorical: the first, second, third, and fourth quartile, unknown	
Smoking status	Categorical: former, current	1992, 1997, or 1999 survey
Pack-years	Continuous	1992 survey
Passive smoke exposure	Personal history of indoor secondhand smoke exposure	1992 survey
	Categorical: yes, no	
Hours since last meal	Categorical: $<$ 2 hours ago, 2-4 hours ago, $\geq$ 4 hours ago, unknown	Survey at blood draw
Year of blood draw	Categorical: 1998, 1999, 2000, 2001	At blood draw

Table S4. The number of proteins associated with individual air pollutants or the air pollution mixture exposure levels at different thresholds. The total number of proteins included in the analyses was 484.

**Air pollution exposure metrics**	**Threshold**	
	unadjusted $P < 0.05$	BH-corrected FDR < 0.2
$PM_{2.5}$	23	0
PM_{10}	48	8
O_3	47	2
NO_2	46	6
SO_2	45	0
CO	36	1
Mixture	22	0

Note: $PM_{2.5}$, fine particulate matter; PM_{10}, coarse particulate matter; NO_2, nitrogen dioxide; O_3, daily 8h maximum ozone; SO_2, sulfur dioxide; CO, carbon monoxide; Mixture, air pollution mixture; BH, Benjamini-Hochberg procedure; FDR, false discovery rate.

Table S5. Summary of the interaction between each air pollutant and smoking status (former/current).

Category	$PM_{2.5}$	PM_{10}	NO_2	O_3	SO_2	CO
$\beta_{pollutant}.\ P < 0.05$ & $\beta_{smoking}.\ P < 0.05$ & $\beta_{interaction}.\ P < 0.05$	2	2	0	19	3	0
$\beta_{pollutant}.\ P < 0.05$ & $\beta_{interaction}.\ P < 0.05$	0	0	2	1	3	0
$\beta_{smoking}.\ P < 0.05$ & $\beta_{interaction}.\ P < 0.05$	4	12	11	20	18	13
$\beta_{interaction}.\ P < 0.05$	0	1	5	2	12	3

Note: $\beta_{pollutant}.\ P$, the P-value for the effect estimate of air pollutant in individual air pollutant-protein model; $\beta_{smoking}.\ P$, the P-value for the effect estimate of smoking status (former/current) in individual air pollutant-protein model; $\beta_{interaction}.\ P$, the P-value for the effect estimate of interaction term between air pollutant and smoking status (former/current) in individual air pollutant-protein model; $PM_{2.5}$, fine particulate matter; PM_{10}, coarse particulate matter; NO_2, nitrogen dioxide; O_3, daily 8h maximum ozone; SO_2, sulfur dioxide; CO, carbon monoxide.

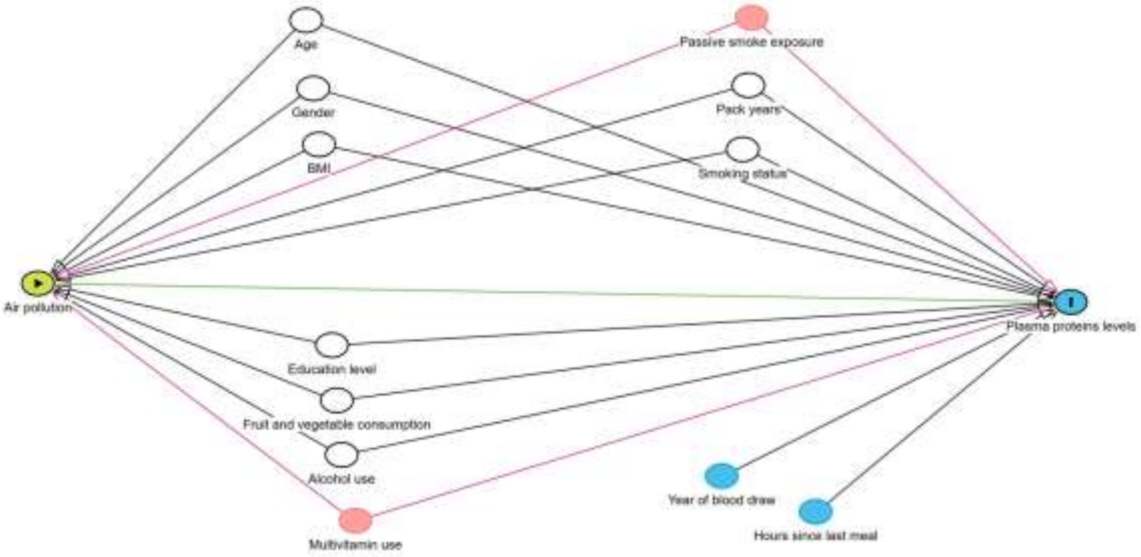

Figure S1. A directed acyclic graph (DAG) of relationships among air pollution exposure, plasma protein levels, and covariates. We identified a list of potential covariates based on a comprehensive literature review and the usage of a DAG. To mitigate the risk of compromising statistical power and over-adjustment, we applied stepwise selection models to determine the final list of covariates. White circles indicate covariates included in the main analyses, while pink and blue circles indicate covariates not included based on stepwise selection.

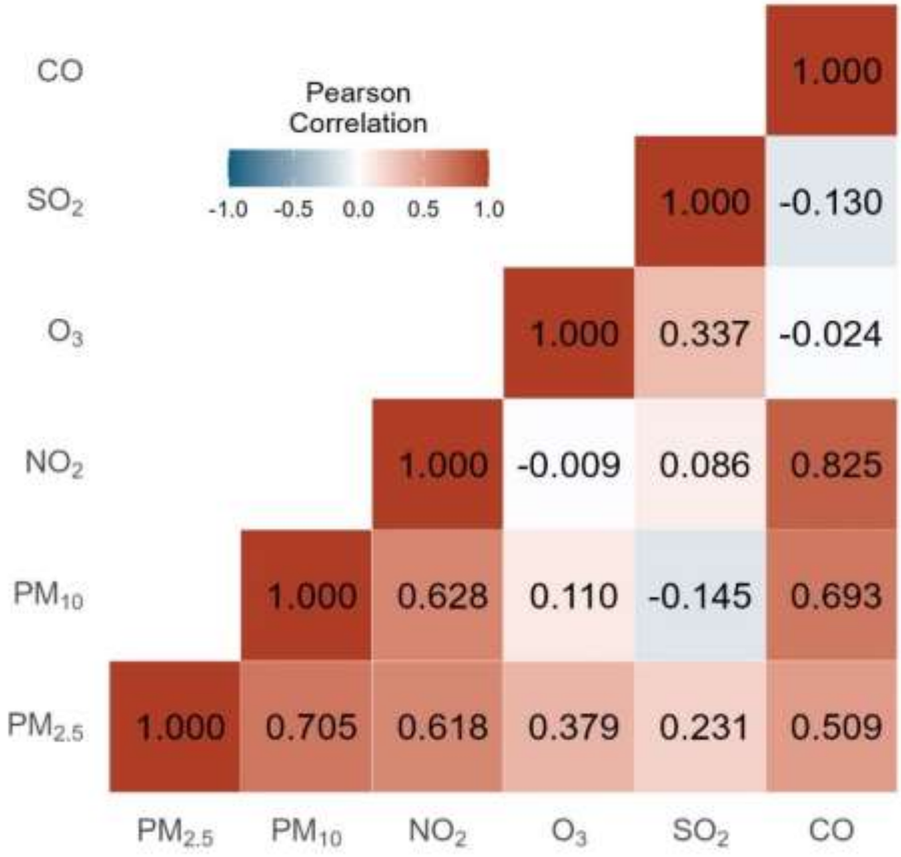

Figure S2. Pearson correlations among annual average levels of six air pollutants.

Note: $PM_{2.5}$, fine particulate matter; PM_{10}, coarse particulate matter; NO_2, nitrogen dioxide; O_3, daily 8h maximum ozone; SO_2, sulfur dioxide; CO, carbon monoxide.

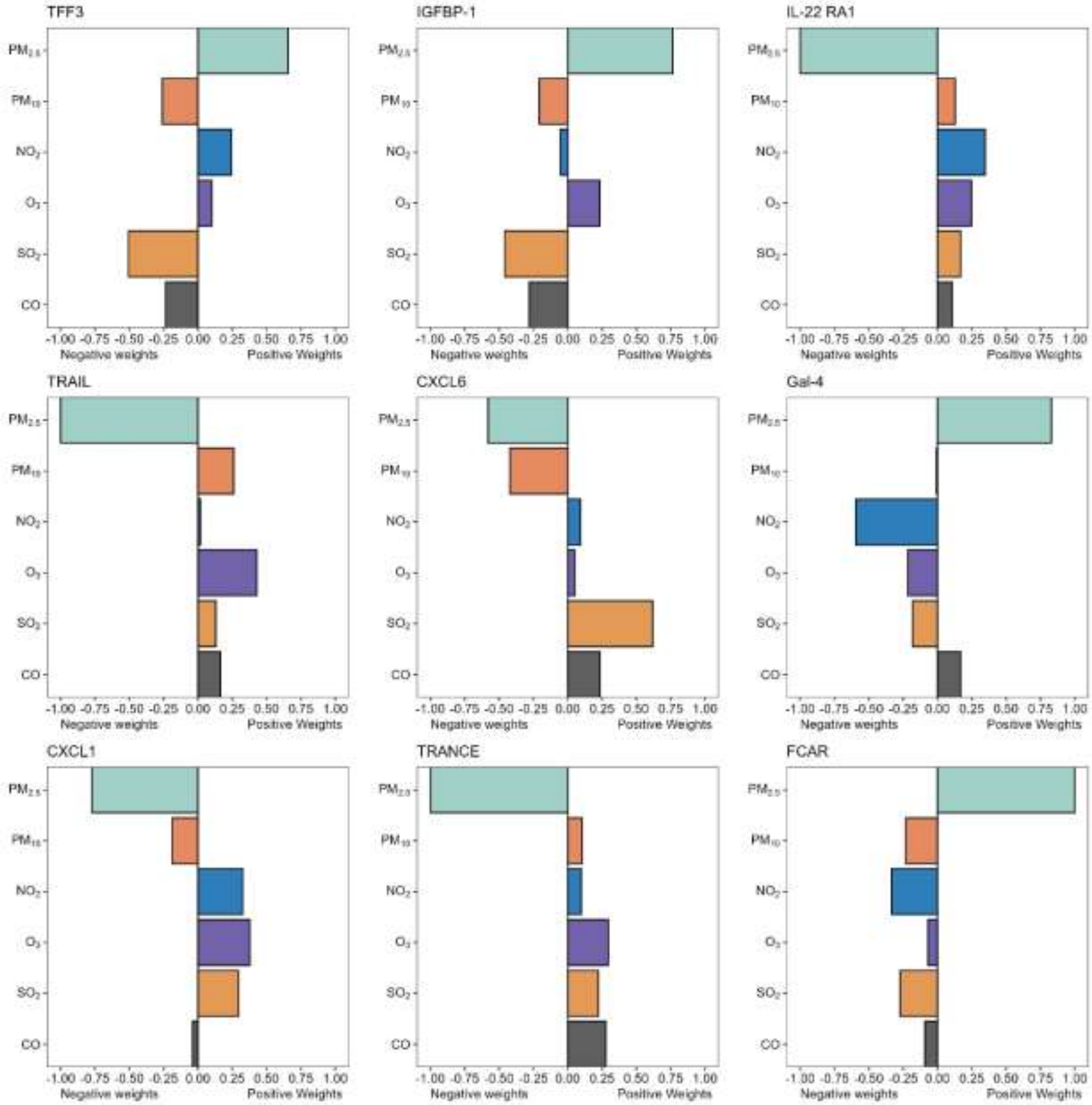

TFF3
IGFBP-1
IL-22 RA1
TRAIL
CXCL6
Gal-4
CXCL1
TRANCE
FCAR
PM2.5
PM10
NO2
O3
SO2
CO
-1.00 -0.75 -0.50 -0.25 0.00 0.25 0.50 0.75 1.00
Negative weights
Positive Weights

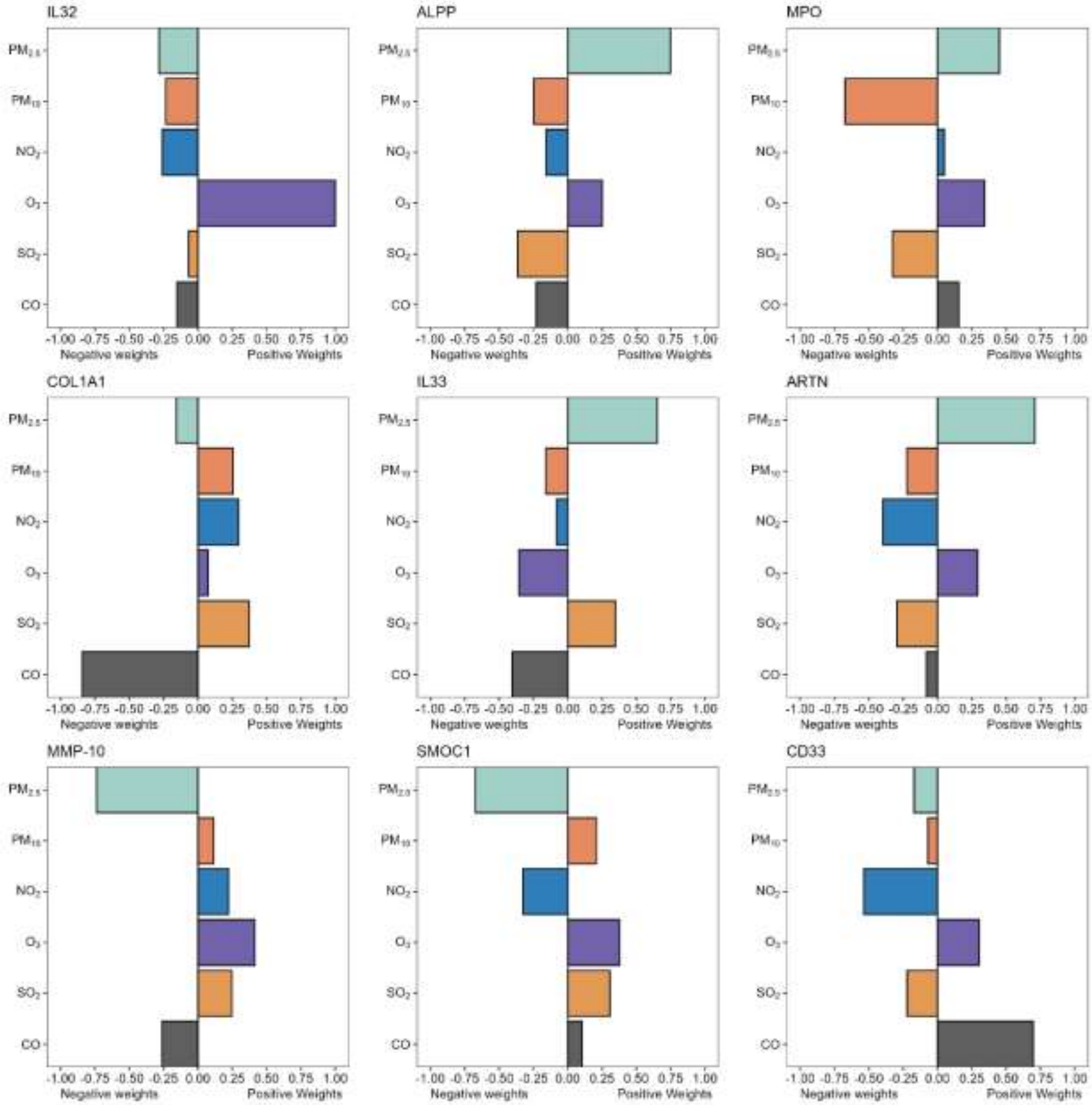
IL32
ALPP
MPO
COL1A1
IL33
ARTN
MMP-10
SMOC1
CD33
PM2.5
PM10
NO2
O3
SO2
CO
-1.00 -0.75 -0.50 -0.25 0.00 0.25 0.50 0.75 1.00
Negative weights
Positive Weights

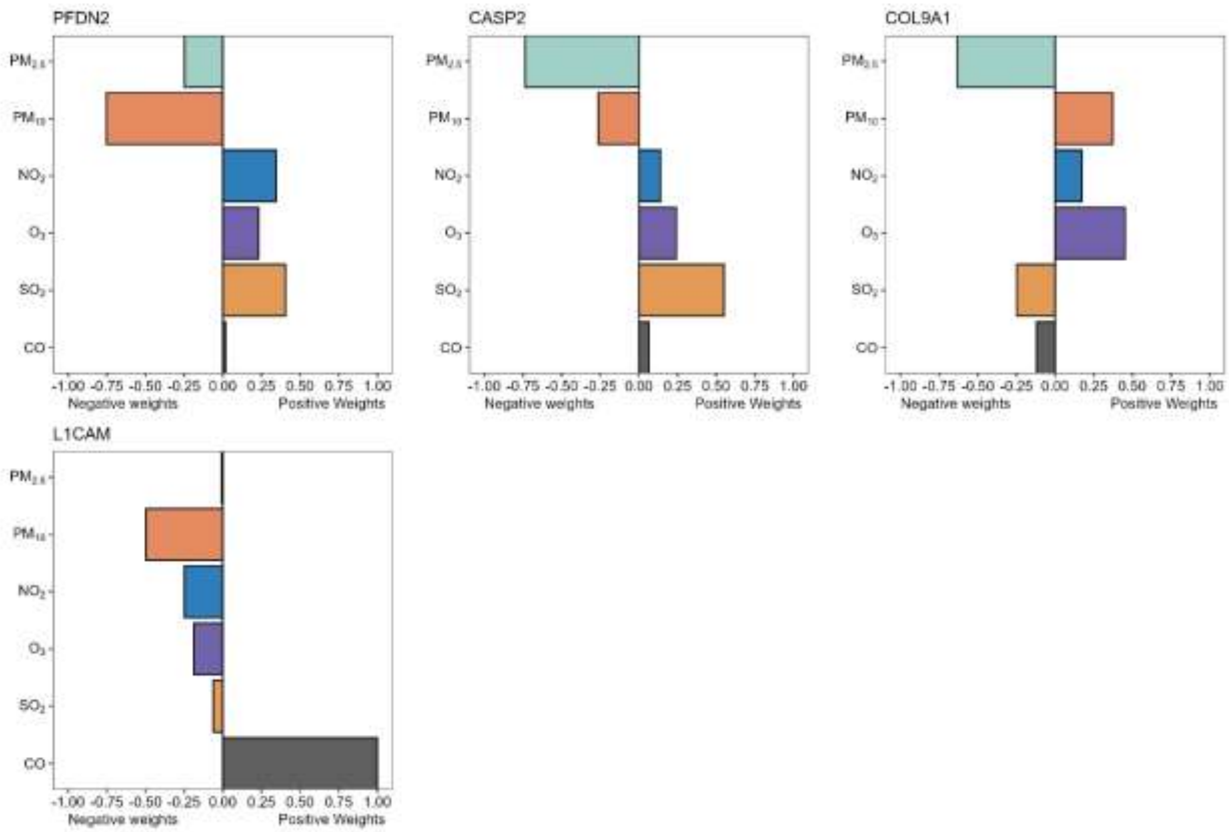

Figure S3. Weights of individual air pollutants for proteins identified in air pollution mixture-protein models (unadjusted $P < 0.05$). The weights represent the proportion of positive or negative partial effects on the overall mixture. The sum for positive weights is one, while the sum for negative weights is negative one. The positive and negative weights cannot be compared directly. The proteins are ordered by unadjusted *P*-value.

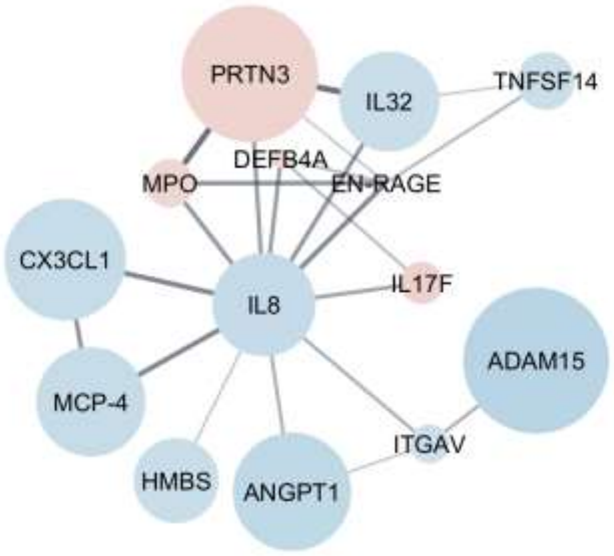
PRTN3
IL32
TNFSF14
DEFB4A
MPO
EN-RAGE
CX3CL1
IL8
IL17F
ADAM15
MCP-4
ITGAV
HMBS
ANGPT1

(A) $PM_{2.5}$

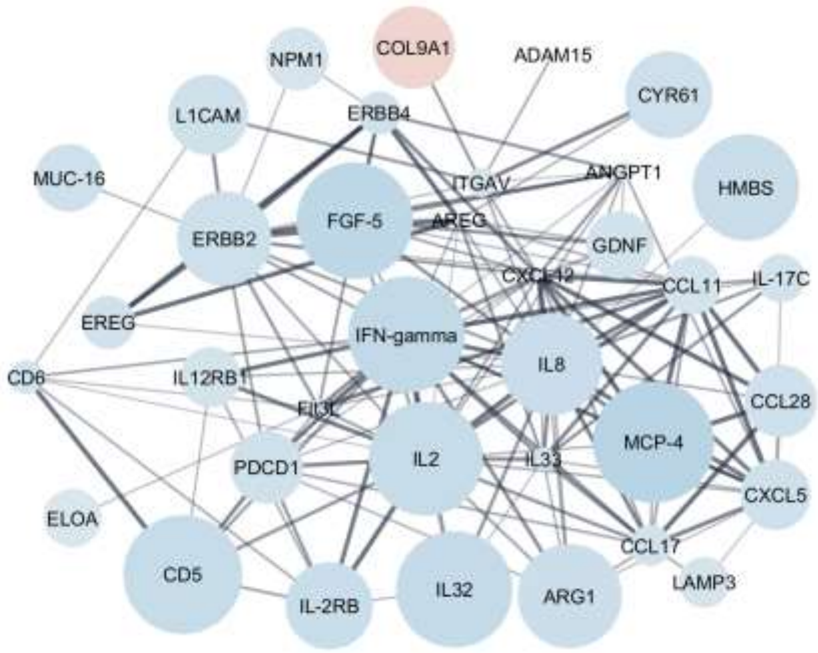
COL9A1
NPM1
ADAM15
CYR61
L1CAM
ERBB4
MUC-16
ITGAV
ANGPT1
HMBS
ERBB2
FGF-5
AREG
GDNF
CXCL12
CCL11
IL-17C
EREG
IFN-gamma
IL8
CD6
IL12RB1
Flt3L
CCL28
MCP-4
PDCD1
IL2
IL33
CXCL5
ELOA
CCL17
LAMP3
CD5
IL-2RB
IL32
ARG1

(B) PM_{10}

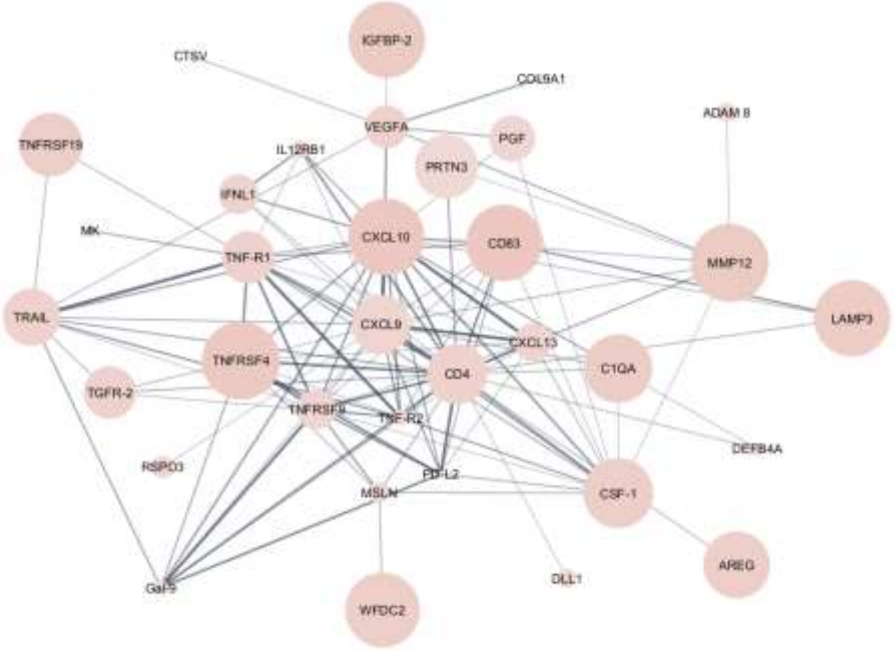
IGFBP-2
CTSV
COL9A1
VEGFA
ADAM 8
PGF
TNFRSF19
IL12RB1
PRTN3
IFNL1
MK
CXCL10
CD83
MMP12
TNF-R1
TRAIL
CXCL9
LAMP3
CXCL13
TNFRSF4
C1QA
CD4
TGFR-2
TNFRSF9
TNF-R2
DEFB4A
RSPO3
PD-L2
MSLN
CSF-1
AREG
DLL1
Gal-9
WFDC2

(C) O_3

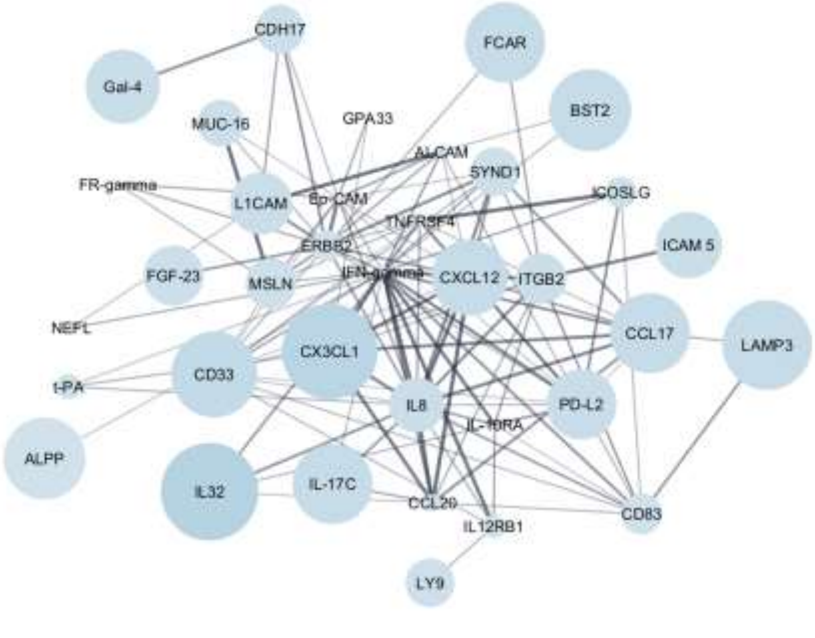
CDH17
FCAR
Gal-4
BST2
MUC-16
GPA33
ALCAM
SYND1
FR-gamma
L1CAM
Ep-CAM
ICOSLG
TNFRSF4
ERBB2
ICAM 5
FGF-23
MSLN
IFN-gamma
CXCL12
ITGB2
NEFL
CCL17
LAMP3
CX3CL1
CD33
t-PA
IL8
PD-L2
IL-10RA
ALPP
IL32
IL-17C
CCL20
IL12RB1
CD83
LY9

(D) NO_2

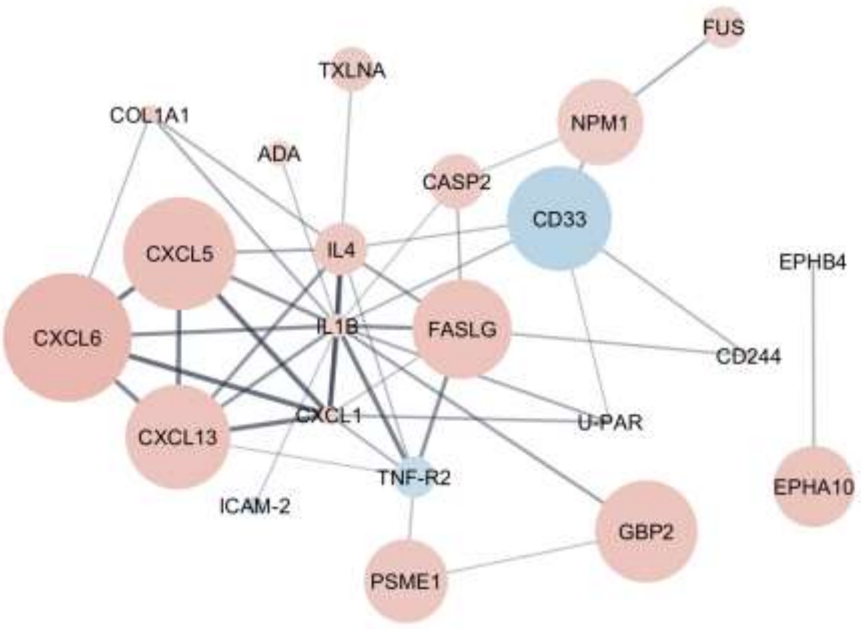

FUS
TXLNA
NPM1
COL1A1
ADA
CASP2
CD33
CXCL5
IL4
EPHB4
CXCL6
IL1B
FASLG
CD244
CXCL1
U-PAR
CXCL13
TNF-R2
EPHA10
ICAM-2
GBP2
PSME1

(E) SO_2

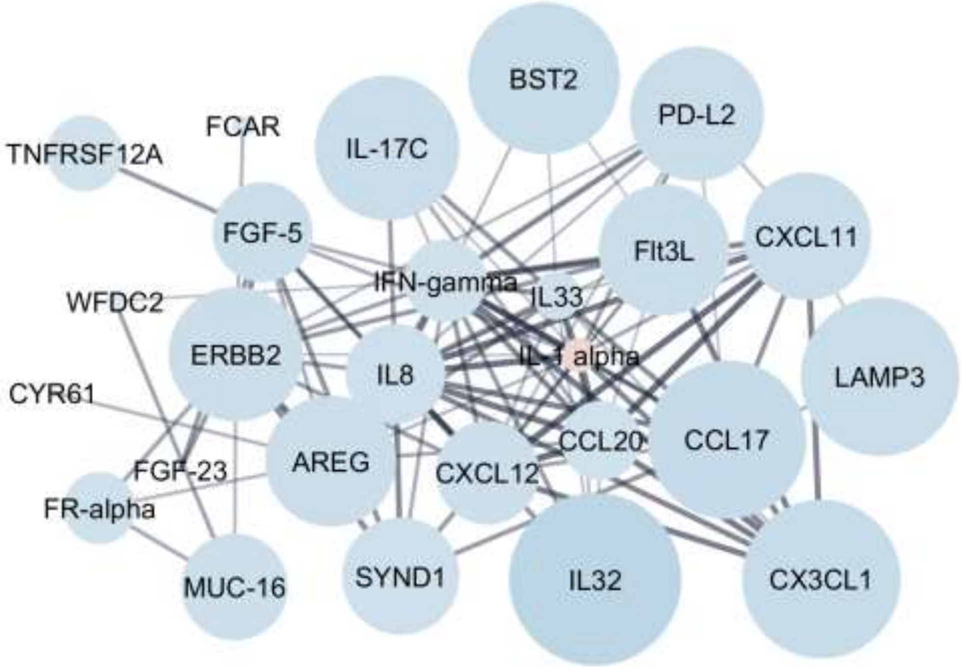

BST2
PD-L2
FCAR
TNFRSF12A
IL-17C
FGF-5
Flt3L
CXCL11
IFN-gamma
IL33
WFDC2
ERBB2
IL-1 alpha
IL8
LAMP3
CYR61
CCL20
CCL17
AREG
FGF-23
CXCL12
FR-alpha
MUC-16
SYND1
IL32
CX3CL1

(F) CO

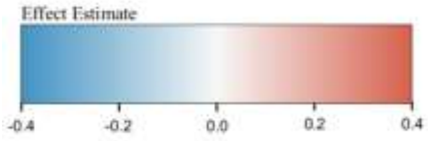

Figure S4. Protein-protein interaction network of individual air pollutant-associated proteins. The proteins of interest were those associated with air pollution at unadjusted $P < 0.05$ from the individual air pollutant models. Larger circles represent smaller P-values. The red color represents a positive association between air pollution metrics and processed level of protein, while blue represents a negative association between air pollution metrics and processed level of protein. The darker the color, the larger the effect estimates. The lines represent the connections between proteins. The thicker the line, the stronger the evidence supporting that connection. The effect estimates were expressed as changes in standardized log_2-transformed levels of proteins per 1/2 interquartile range increase in air pollutant levels, controlling for covariates.

Note: $PM_{2.5}$, fine particulate matter; PM_{10}, coarse particulate matter; NO_2, nitrogen dioxide; O_3, daily 8h maximum ozone; SO_2, sulfur dioxide; CO, carbon monoxide.

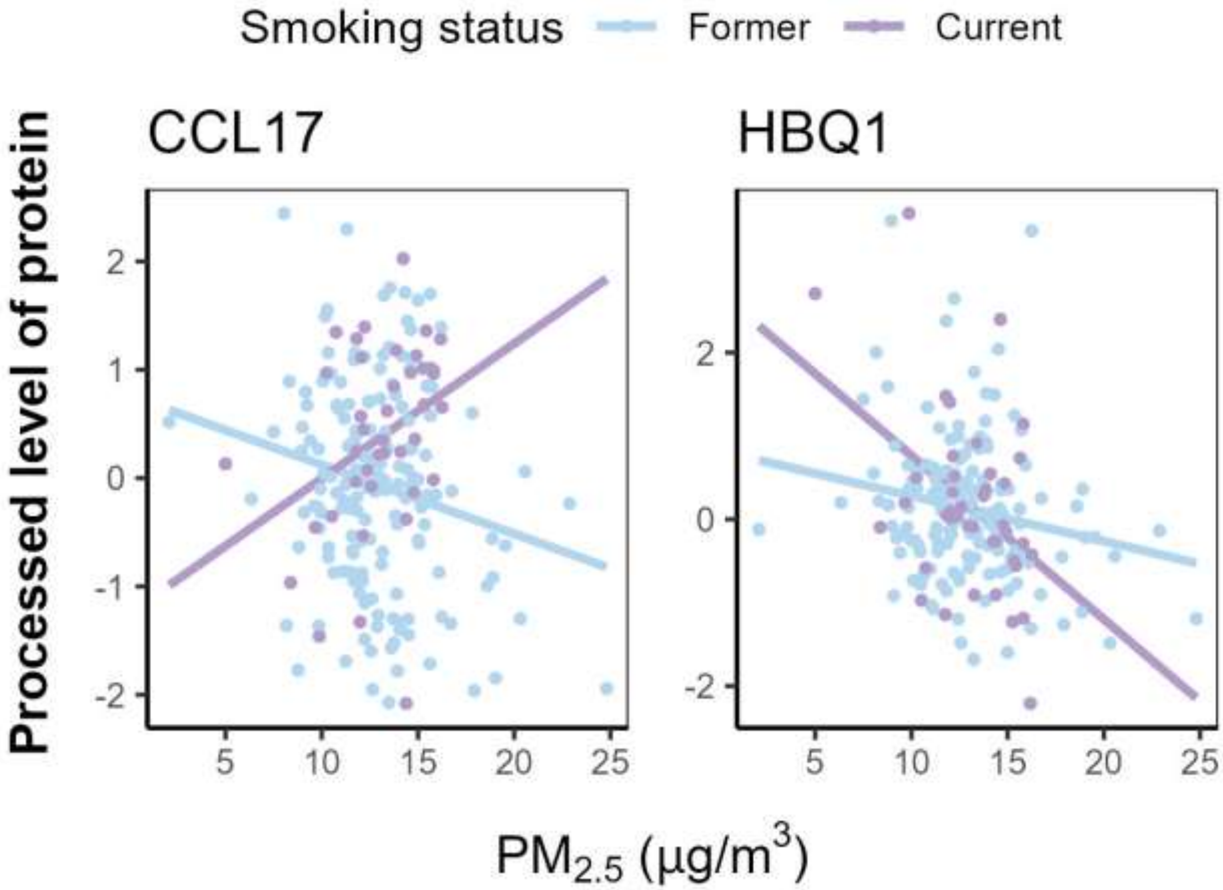
Smoking status
Former
Current
CCL17
HBQ1
Processed level of protein
2
1
0
-1
-2
5
10
15
20
25
PM2.5 (μg/m3)

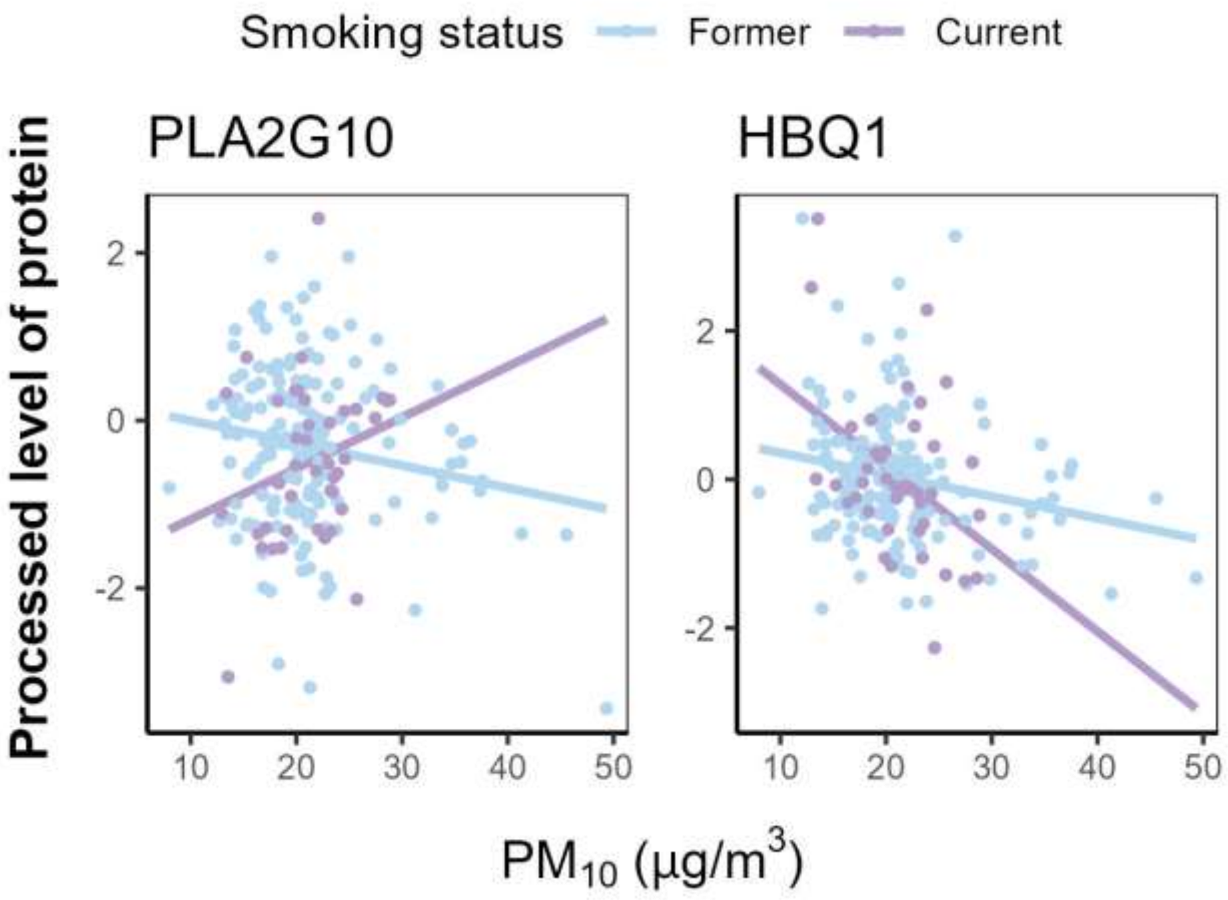
Smoking status
Former
Current
PLA2G10
HBQ1
Processed level of protein
2
0
-2
10
20
30
40
50
PM10 (µg/m3)

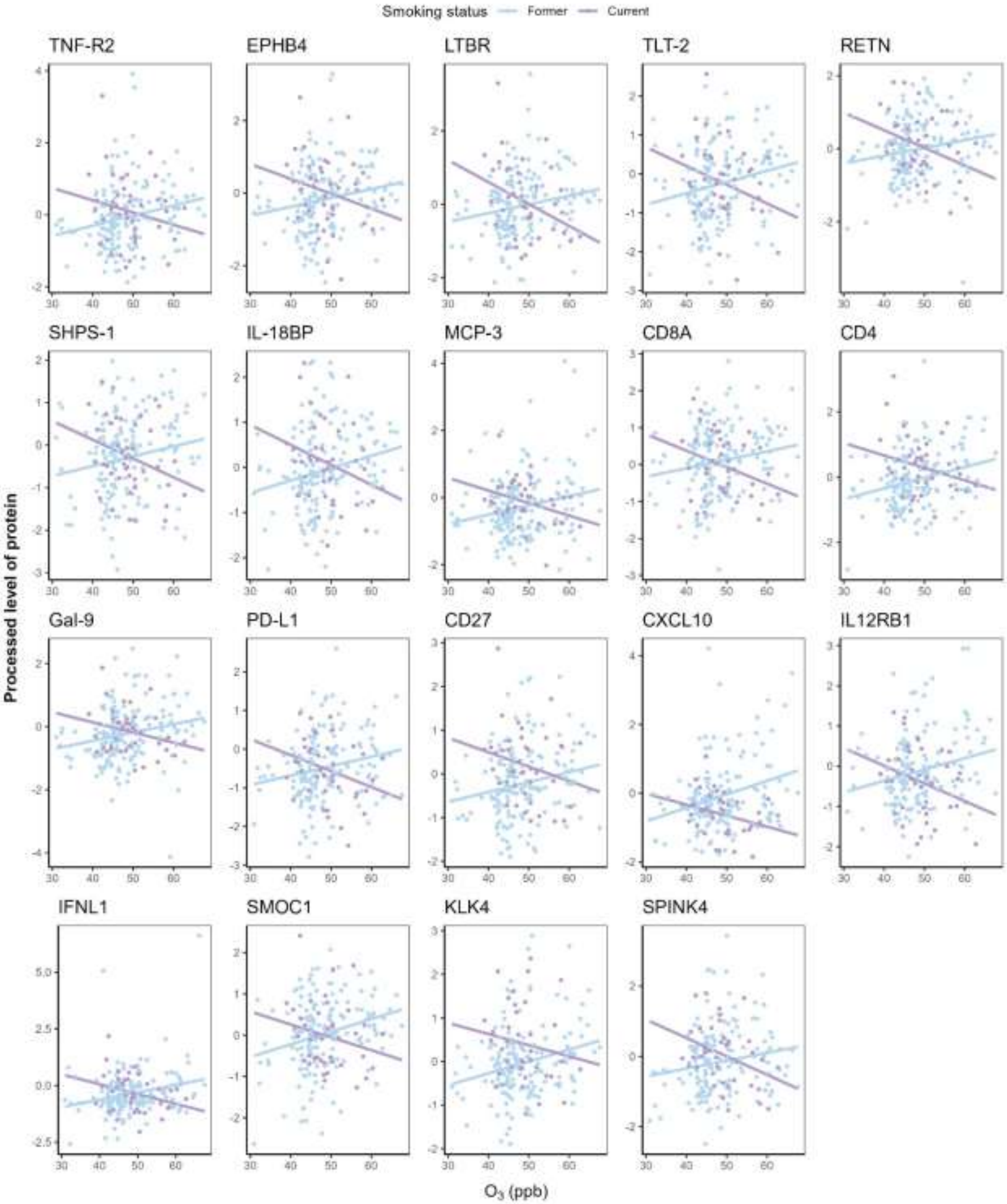
Smoking status
Former
Current
TNF-R2
EPHB4
LTBR
TLT-2
RETN
SHPS-1
IL-18BP
MCP-3
CD8A
CD4
Gal-9
PD-L1
CD27
CXCL10
IL12RB1
IFNL1
SMOC1
KLK4
SPINK4
Processed level of protein
O3 (ppb)

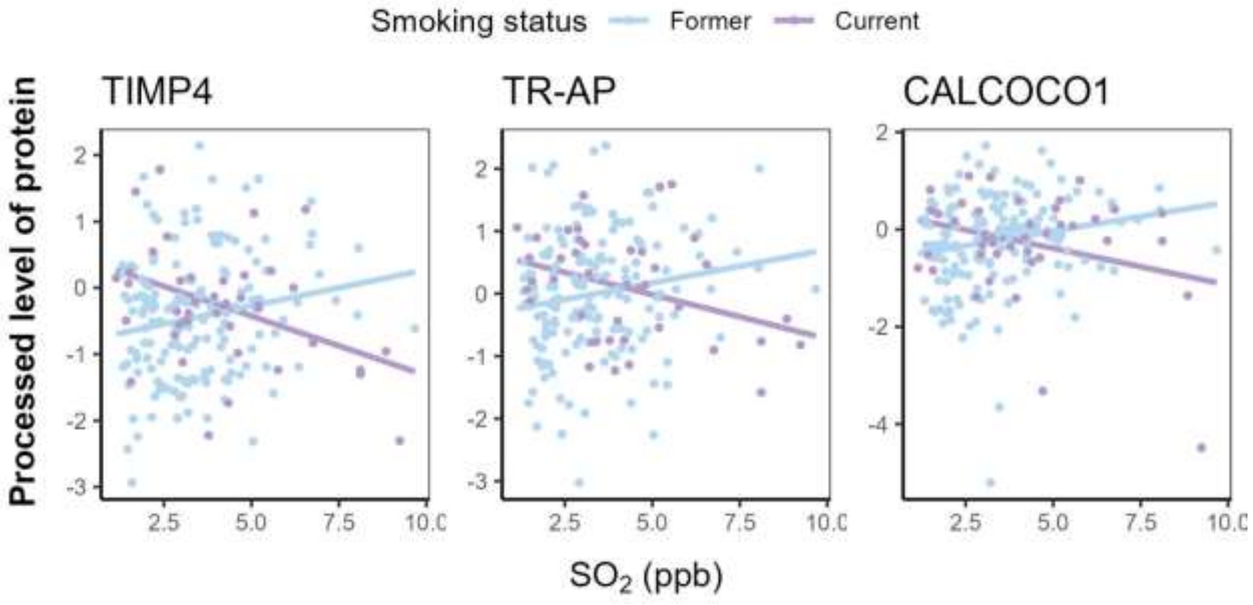

Figure S5. The plots for the effects of air pollutants on proteins by smoking status, holding all other variables constant. Only proteins where the effect estimates of air pollutants, smoking status (former/current smoker), and interaction term in individual air pollutant-protein models were significant (unadjusted $P < 0.05$) were shown.

Reference

1. Manes, N. P.; Song, J.; Nita-Lazar, A., EnsMOD: A Software Program for Omics Sample Outlier Detection. *J Comput Biol* **2023,** *30* (6), 726-735.
2. Chen, X.; Zhang, B.; Wang, T.; Bonni, A.; Zhao, G., Robust principal component analysis for accurate outlier sample detection in RNA-Seq data. *BMC bioinformatics* **2020,** *21*, 1-20.
3. Selicato, L.; Esposito, F.; Gargano, G.; Vegliante, M. C.; Opinto, G.; Zaccaria, G. M.; Ciavarella, S.; Guarini, A.; Del Buono, N., A new ensemble method for detecting anomalies in gene expression matrices. *Mathematics* **2021,** *9* (8), 882.
4. Hubert, M.; Rousseeuw, P. J.; Vanden Branden, K., ROBPCA: A New Approach to Robust Principal Component Analysis. *Technometrics* **2005,** *47* (1), 64-79.
